# New Class of Tunable Choline Bromide-Based Hydrophobic Deep Eutectic Solvents for the Extraction of Bioactive Compounds of Varying Polarity from a Plant Matrix

**DOI:** 10.1021/acssuschemeng.3c00185

**Published:** 2023-04-20

**Authors:** Giulia Mastellone, Nabeel Mujtaba Abbasi, Cecilia Cagliero, Jared L. Anderson

**Affiliations:** † Department of Drug Science and Technology, University of Turin, Via Pietro Giuria, 9, I-10125 Torino, Italy; ‡ Ames National Laboratory—USDOE and Department of Chemistry, Iowa State University, 1605 Gilman Hall, 50011 Ames, Iowa, United States

**Keywords:** hydrophobic deep eutectic solvents, choline chloride, extraction, Cannabis sativa L., bioactive compounds, liquid chromatography

## Abstract

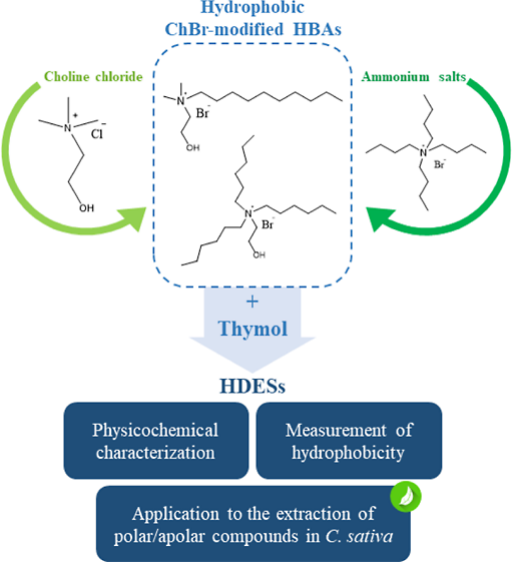

Deep eutectic solvents (DESs) are a class of sustainable solvents that have found numerous applications in different fields. One of their main attributes is the possibility of easily modifying their physicochemical properties by varying the type of hydrogen bond donor (HBD) and hydrogen bond acceptor (HBA) that comprise them. Choline chloride ([Ch^+^][Cl^–^])-based hydrophilic DESs were among the first studied and the most used because of their capacity to easily create a hydrogen bonding network that lies in its unique chemical structure, characterized by a hydroxyl substituent within the ammonium headgroup. In this study, a new class of hydrophobic [Ch^+^][Br^–^]-modified salts were synthesized to produce HBAs with similar properties to choline for the preparation of hydrophobic DESs. Six different [Ch^+^][Br^–^]-based HDESs were prepared and characterized in terms of hydrophobicity, viscosity, and solvation properties (hydrogen bonding, dispersion, dipolarity/polarizability, n−π, and π–π interactions). They were employed as solvents in a microextraction method for the determination of phytochemicals in *Cannabis sativa* L. plant. The extraction performance of the [Ch^+^][Br^–^]-based HDESs was compared to eutectic mixtures based on conventional hydrophobic HBAs, and the results revealed that the unique properties of [Ch^+^][Br^–^]-modified salts allowed for the extraction of both hydrophilic (i.e., flavonoids) and hydrophobic compounds (i.e., cannabinoids).

## Abstract 2

A new class of tunable [Ch^+^][Br^−^]-based HDESs are synthesized and used for the extraction of bioactive compounds of varying polarity from *Cannabis sativa* L.

## Introduction

In recent years, deep eutectic solvents (DESs) have been more widely used by the scientific community due to their desirable properties that include negligible vapor pressure, low cost of raw materials, and possibility to easily vary their composition. They consist of a mixture of two or more components able to form a hydrogen bonding network, resulting in a lower melting point compared to the starting materials. Hydrophilic or hydrophobic DESs can be easily prepared depending on the chemical structures of the hydrogen bond acceptor (HBA) and hydrogen bond donor (HBD) employed. Several studies^1−4^ have shown DESs to be effective solvents for the extraction of natural compounds from matrices of vegetal origin prior to downstream chromatographic analysis. Plants are complex samples characterized by several compounds belonging to different chemical classes, divided into primary (e.g., amino acids and polysaccharides) and secondary (e.g., terpenes and phenolics) metabolites.^5^ Forms of sample preparation, which include processes of homogenization, cleanup of the matrix, and enrichment of the target analytes, are therefore necessary to make the sample compatible with downstream analysis. These pre-treatment steps have a significant impact on the sustainability of the overall analysis process as they often require the use of disposable materials, energy-consuming equipment and instrumentation, and extraction methods that employ harmful solvents.^6^ Thus, great efforts have been made by the scientific community to develop functional methods aimed at improving the greenness of the extraction processes. In this context, new classes of extraction phases can be adopted to overcome the limits of conventional solvents. A number of greener solvents and materials have been proposed, most of them with the advantage of having a chemical structure that can be tailored to increase the selectivity toward specific compounds.^7,8^ For instance, hydrophobic DESs (HDESs) can be easily prepared by mixing HBAs and HBDs featuring low water solubility, such as alcohols, carboxylic acids, and natural phenolics and terpenoids. Since they are not miscible with water, they can be easily recovered from aqueous samples, and their applications are gaining ground within the analytical field.

Choline chloride ([Ch^+^][Cl^–^])-based DESs were among the first eutectic mixtures reported by Abbott et al. in 2003.^9^ They are the most studied types of DESs due to their relative biodegradability and the low toxicity of [Ch^+^][Cl^–^]. Moreover, [Ch^+^][Cl^–^] can form DESs with a wider variety of HBDs, contrary to other tetraalkylammonium salts. The chemical structure of [Ch^+^][Cl^–^] features a hydroxyl group on one of the alkyl chain substituents, which is not present in the structure of other tetraalkylammonium salts. Migliorati et al.^10^ compared through molecular dynamics simulations the arrangement of the hydrogen bonding network when [Ch^+^][Cl^–^] and butyltrimethylammonium chloride are mixed with urea at a 1:2 molar ratio. The unique three-dimensional structure of these DESs affects the melting point depression, which is larger for [Ch^+^][Cl^–^]-based DES due to more favorable interactions. However, the hydrophilic nature of [Ch^+^][Cl^–^] makes it a less favorable candidate for HDES preparation. A number of less hydrophilic salts are commercially available and have been used to prepare HDESs, such as tetrabutylammonium bromide/chloride, tetraoctylammonium bromide/chloride, and ethyltrioctylammonium bromide/chloride.^11−13^ Contrary to [Ch^+^][Cl^–^], these alkylammonium salts do not feature the hydroxyl group on one of the alkyl chain substituents which, as mentioned above, is important for the formation of the hydrogen bonding network.

Environmental sustainability is currently a topic of global interest, and regulatory organizations are developing policies to encourage the reduction of waste and pollution and the efficient use of resources. Plant and plant-based products represent a sustainable source of bioactive compounds which can be exploited for feed, functional food, and food supplement industries.^14^ Due to the relatively easy cultivation of *Cannabis* sp., this crop is now the subject of renewed interest in many fields, including textiles, nutraceuticals, chemical and energy, among others. Moreover, its rich phytocomplex, which includes several bioactive secondary metabolites, can be exploited in the pharmaceutical and cosmetic industries. Fiber-type *C. sativa* L., also called hemp or industrial hemp, represents an interesting multifunctional crop for industrial purposes due to the low content of the psychotomimetic compound delta-9-tetrahydrocannabidiol (Δ^9^-THC) that cannot exceed 0.2% in dry matter, according to the European Industrial Hemp Association.^15^

The main goal of this study was to obtain a novel class of hydrophobic compounds to be used in the preparation of HDESs. The chemical structure design of these new compounds was guided according to the following features: (i) low solubility in water and (ii) capacity to form a hydrogen bonding network. The structure of choline was employed as a model, and the length of alkyl chain substituents appended to the ammonium headgroup was increased to improve the hydrophobicity and broaden the range of application of the new compounds. Contrary to other commercial ammonium salts, the hydroxyl functional group feature of choline was maintained, thus improving the capacity of the new compounds to form hydrogen bonding. Synthesis was centered around [Ch^+^][Br^–^] derivatives as they exhibited lower solubility in water compared to [Ch^+^][Cl^–^] derivatives. The salts were mixed with thymol to form six different [Ch^+^][Br^–^]-based HDESs that were thoroughly and systematically characterized in terms of hydrophobicity, viscosity, and their ensuing solvation properties. In the second part of the study, the suitability of [Ch^+^][Br^–^]-based HDESs as extraction solvents for the determination of phytochemicals of varying polarity in *Cannabis sativa* L. was tested using a dispersive solid–liquid microextraction (DSLME) method.^16^ DSLME was optimized, and the extraction performance of the six [Ch^+^][Br^–^]-based HDESs was compared with eutectic mixtures based on commercial salts.

## Experimental Section

### Materials

HPLC-grade acetonitrile, methanol (MeOH) (>99.9% purity), acetone (>99.0% purity), formic acid (>98.0% purity), diethyl ether (>99.0% purity), ethyl acetate (>99.5% purity), and hexane (>98.5% purity) were supplied by Merck Life Science S.r.l. (Milan, Italy). Deionized water (18.2 MΩ cm) was obtained from a Milli-Q purification system (Millipore, Bedford, MA, USA). A deactivated fused silica capillary was procured from MEGA (Legnano, Milano, Italy). The reagents tripropylamine, trihexylamine, trioctylamine, dimethylaminoethanol, 2-bromoethanol, 1-bromohexane, 1-bromodecane, 1-bromododecane, 1-bromotetradecane, and 1-bromohexadecane, obtained from Merck Life Science S.r.l, were used in the synthesis of [Ch^+^][Br^–^]-modified salts. Tetrabutylammonium bromide and tetraoctylammonium bromide were selected as reference commercial salts and were provided by Merck Life Science S.r.l. Individual stock solutions of cannabidiolic acid, CBDA CAS 1244-58-2 (Merck Life Science S.r.l), luteolin-7-*O*-glucuronide CAS 29741-10-4, and apigenin-7-*O*-glucuronide CAS 29741-09-1 (Phytolab, Vestenbergsgreuth, Germany) were prepared in MeOH 100% and MeOH 85% for luteolin-7-*O*-glucuronide at 1 mg mL^–1^. A standard working solution containing all analytes was prepared in MeOH by dilution of the stock solutions to a concentration of 0.1 mg mL^–1^. These solutions were kept protected from light and refrigerated at −21 °C. For the preparation of [Ch^+^][Br^–^]-based HDESs, thymol was purchased from Merck Life Science S.r.l. For NMR analysis, dimethyl sulfoxide (DMSO-*d*_6_) and chloroform (CDCl_3_) were procured from Cambridge Isotope Laboratories (Andover, MA, USA). The plant samples used in this study (fiber-type *C. sativa* L.) were kindly provided by the Institute of Science of Food Production, National Research Council (Grugliasco, Italy). Hemp plants were grown in the Western Po Valley (Italy), and the aerial parts (mainly stem and leaves) were collected before flowering. The harvested samples were immediately freeze-dried and ground to a fine powder and passed through a 1 mm screen with a Cyclotec mill (Tecator, Herndon, VA, USA). They were stored at 4 °C to prevent degradation.

### Synthesis of [Ch^+^][Br^–^]-Modified Salts

**Figure 1:**
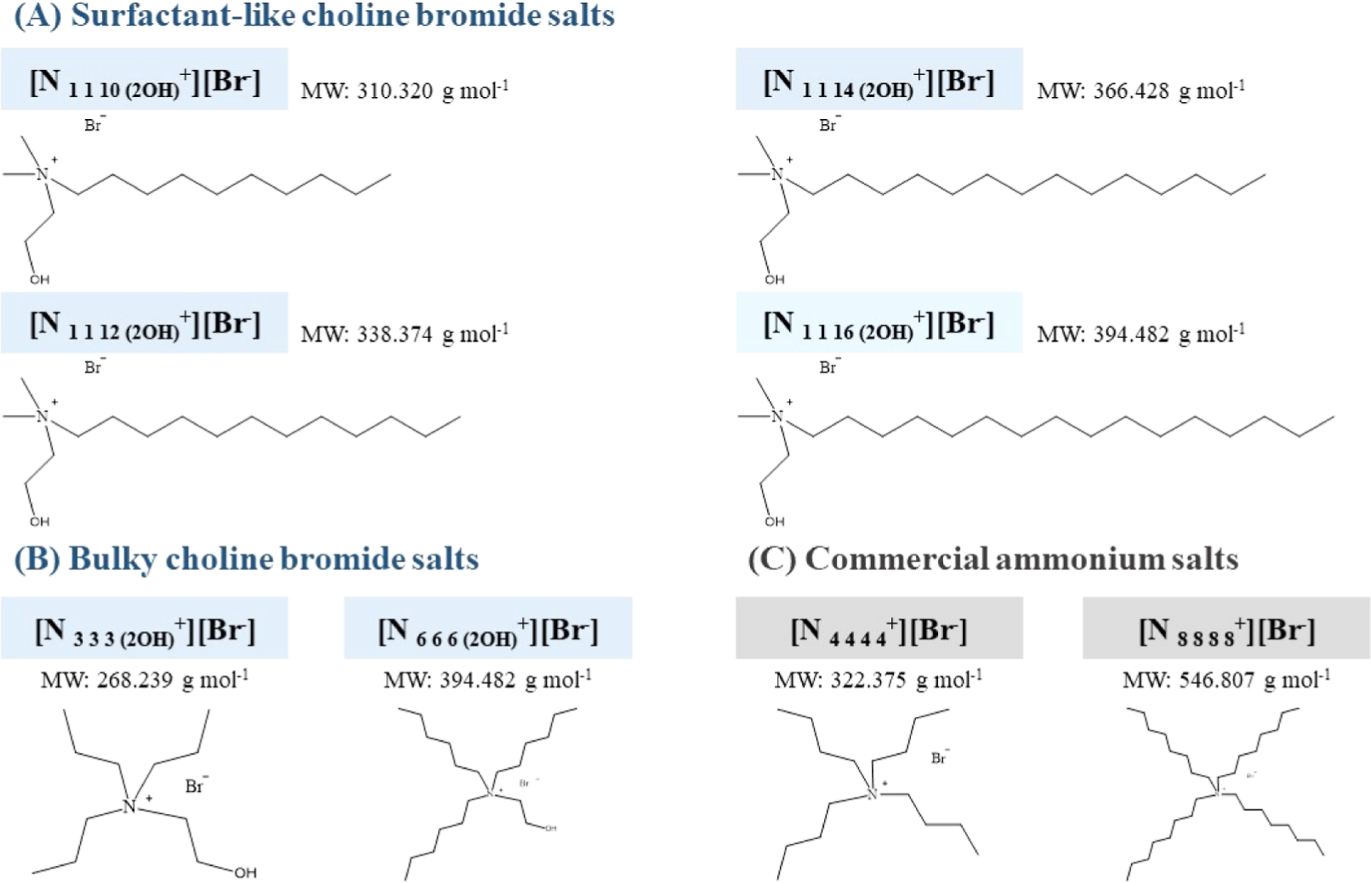
Chemical structures and molecular weight of the salts used in this study, where groups (A,B) were obtained by chemical synthesis and group (C) was used for comparison purposes.

Six [Ch^+^][Br^–^]-modified salts were synthesized to obtain hydrogen bond acceptors (HBAs) with hydrophobic features. Two main classes of ammonium salts were obtained: (A) [N_*x x x* (2OH)_^+^][Br^–^] and (B) [N_1 1 *y* (2OH)_^+^][Br^–^], where *x* = 3 or 6 and *y* = 10, 12, 14, or 16. Moreover, two commercial ammonium salts, tetrabutylammonium bromide and tetraoctylammonium bromide, were used for comparison purposes. Figure 1 shows the structures of salts used in the current study, while Table S1 of the Supporting Information summarizes the main reaction conditions for every salt.

For the first group (A), the synthesis protocol was modeled based on that reported by Dupont et al.,^17^ with some modifications, and the purity was confirmed by ^1^H and ^13^C NMR. Briefly, amine and bromoethanol were dissolved in toluene and allowed to react under reflux for 48 h at 110 °C. After cooling, the solvent was removed under rotary evaporation, and the product was purified (see Table S1 of the Supporting Information for details) to form a white powder. For the second group (B), the synthesis protocol was based on that reported by Costa et al.^18^ after some modification and purity confirmed by ^1^H and ^13^C NMR. In this case, the alkyl halide and 2-(dimethylamino)ethanol were mixed in hexane and heated to 80 °C under reflux for 8–10 h. The white solid precipitate was then filtered and washed with hexane. All products were dried under reduced pressure and stored in a vacuum oven. The NMR spectra for all synthesized compounds are provided in the Supporting Information.

### Preparation of [Ch^+^][Br^–^]-Based HDESs

The HDESs tested in the study are reported in Table 1 and were prepared according to the heating and stirring method by mixing HBA and HBD for 30 min at 60 °C under magnetic stirring. After obtaining a homogeneous solvent, they were stored in a desiccator to prevent moisture adsorption from the atmosphere.

**Table 1:** HBD, HBAs, and Molar Ratios of the HDESs Examined in This Study

HBA	HBD	molar ratio
[N_1 1 10 (2OH)_^+^][Br^–^]	thymol	1:2
[N_1 1 12 (2OH)_^+^][Br^–^]	thymol	1:2
[N_1 1 14 (2OH)_^+^][Br^–^]	thymol	1:2
[N_1 1 16 (2OH)_^+^][Br^–^]	thymol	1:2
[N_3 3 3 (2OH)_^+^][Br^–^]	thymol	1:2
[N_6 6 6 (2OH)_^+^][Br^–^]	thymol	1:2
[N_4 4 4 4_^+^][Br^–^]	thymol	1:2
[N_8 8 8 8_^+^][Br^–^]	thymol	1:2

### Characterization of [Ch^+^][Br^–^]-Based HDESs

Bruker (Massachusetts, USA) 600 MHz and JEOL ECZR (Massachusetts, USA) 600 MHz NMR spectrometers were used to characterize the purity of the salts and HDESs. To measure the hydrophobicity of the DESs, water content was analyzed by a Metrohm 831 Karl Fischer coulometric titration system (Herisau, Switzerland) before and after mixing the solvent with water at neutral pH and 25 °C. The titration cell was filled with a Hydranal-Coulomat AG reagent, and the contents of the chamber were stirred at 300 rpm. The same amount (500 mg) of HDES and water was gently mixed for 30 s with a spatula to avoid the formation of bubbles and then subjected to centrifugation to facilitate rapid separation of the two phases. Finally, the HDES phase was sampled using a 3 mL Luer lock tip sterile plastic syringe, and its water content was measured. The stability of the eutectic mixtures during the extraction process was also investigated (see the Supporting Information for more details). The solvents under study were subjected to DSLME and then isolated to measure and compare the ^1^H NMR profile with the one obtained prior to extraction.

Differential scanning calorimetry (DSC) was performed on HDESs to investigate their thermal properties using a NETZSCH DSC 214 Polyma instrument (Selb, Germany), and all thermograms were analyzed using NETZSCH Proteus software. The solvatochromic response of fluorescent dyes was studied using a Synergy H1 microplate reader (Biotek, Winooski, Vermont). The thermal properties of select HDESs were characterized using the following protocol: the samples were cooled from 40 to −120 °C at a rate of 10 °C min^–1^ and subsequently heated to 80 °C at the same rate. DSC thermograms were analyzed to obtain the melting peak and/or glass-transition temperatures for DESs, as shown in Table S2.

HDES viscosity was measured using an AMETEK Brookfield DV1 cone and a plate viscometer (Middleborough, MA, USA) equipped with a CPA-51Z cone spindle, at 21.6 °C, and all the data is provided in Table S3. In addition, all DESs were analyzed using attenuated total reflection Fourier transform infrared (ATR–FTIR) spectroscopy and scanned between 4000 and 500 cm^–1^. All IR measurements were performed on a Bruker VERTEX 80 FTIR spectrometer (Billerica, MA, USA); spectra are provided in Figures S1–S9 in the Supporting Information.

To investigate the polarity of hydrophobic [Ch^+^][Br^–^]-based HDESs using the solvatochromic dye method, the dyes 4-nitroaniline (4-NA), Nile red (NR), and *N*,*N*-diethyl-4-nitroaniline (*N*,*N*-DENA) were employed. Each dye was dissolved in methanol at a 0.01 M concentration and refrigerated under 4 °C before transferring 100 μL into a neat Eppendorf tube. A steady stream of nitrogen was passed through the Eppendorf tube to evaporate methanol, leaving behind the solvatochromic dye as a thin film on the walls of the tube. A 100 μL volume of each HDES was transferred into the Eppendorf tube and mixed thoroughly with the dye until a homogeneous mixture was obtained. A 20 μL volume of the resulting mixture was transferred into a 96-well plate to measure its solvatochromic behavior using a microplate reader. UV–vis molecular absorbance data was acquired through spectral scanning of HDES/dye mixtures between wavelengths of 350 and 700 nm in 1 nm increments, and the maximum absorption wavelength (λ_max_) was recorded.^19^ Spectroscopic measurements were conducted in triplicate, and the average of all values was calculated.

Solvation characteristics of DESs were further studied using inverse gas chromatography (IGC) by employing them as GC stationary phases. To prepare DES chromatographic columns using the static method of column preparation, 32 mg of each DES was transferred into a clean 20 mL glass vial and solubilized in 10 mL of dichloromethane. The DES solutions were injected into 5 m segments of a deactivated fused silica capillary, which were then sealed at one end, while the other end was connected to a vacuum, before placing the resulting setup in a water bath maintained at 40 °C. Dichloromethane was gradually and steadily evaporated, leaving behind a uniform thin film of DES coated on the inner walls of the fused silica capillary having an approximate thickness of 0.20 μm.^20^

An Agilent 7890B gas chromatograph (Santa Clara, USA) equipped with a flame ionization detector was employed for IGC experiments. A deactivated fused silica capillary was procured from MEGA (Legnano, Milano, Italy). Prior to performing GC measurements, all columns were placed inside a gas chromatograph and thoroughly conditioned by flowing helium (1 mL min^–1^ constant flow) at 60 °C for 30 min. Inlet/detector temperatures were kept constant at 150 °C, while a 20:1 inlet split ratio was employed. Additionally, IGC measurements were conducted at 40, 50, and 60 °C by systematically varying the GC oven temperature. Column efficiencies were measured by chromatographically separating naphthalene (1 g L^–1^) at 60 °C and were determined to range from 1500 to 2900 plates/meter. Probe molecules comprising volatile organic compounds such as branched and straight-chained alcohols, nitroalkanes, and haloalkanes were dissolved in dichloromethane at the same concentration (1 g L^–1^) and injected onto the DES columns to study their chromatographic retention. A detailed list of 50 probe molecules employed in this study can be found in Table S4.

### [Ch^+^][Br^–^] HDES-Based DSLME

**Figure 2:**
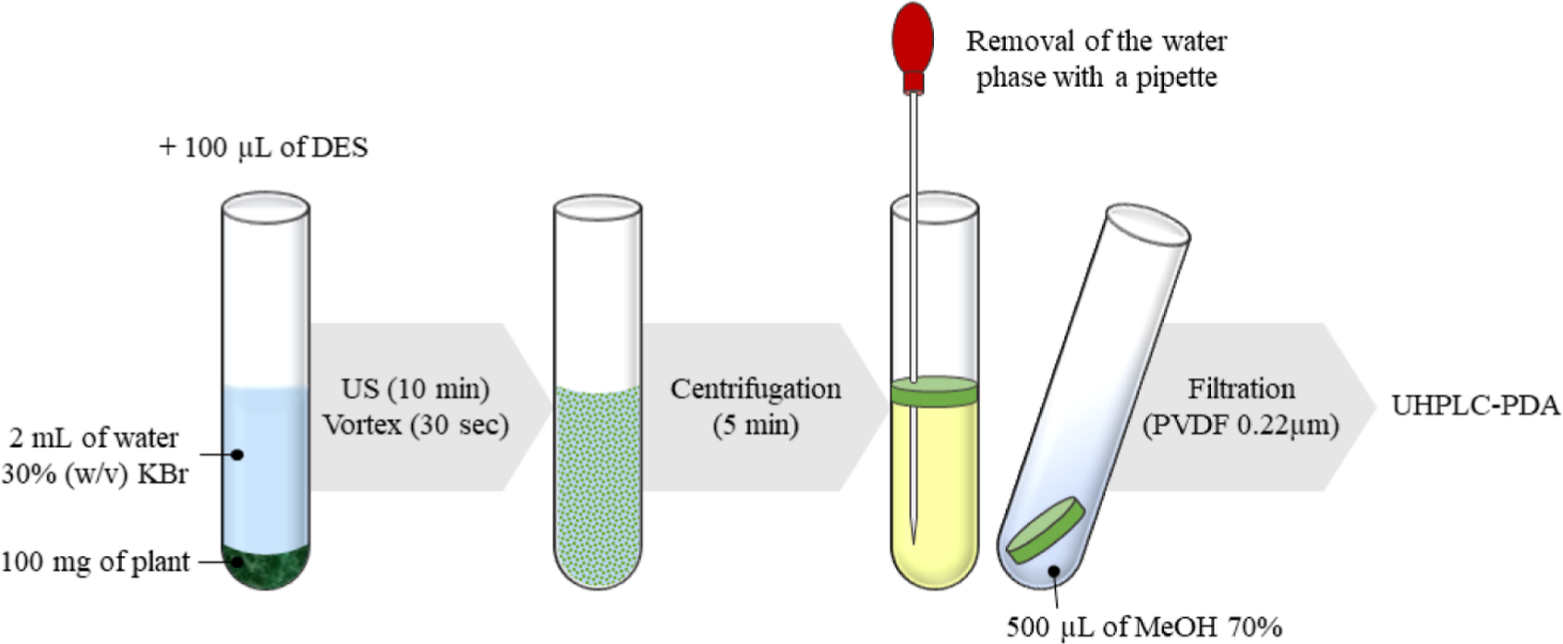
Dispersive solid–liquid microextraction (DSLME) method used for the extraction of phytochemicals from fiber-type *C. sativa* L. aerial parts.

The following equipment were employed for DSLME: a Sonica S3 EP 2400 ultrasonic bath (Soltec, Milan, Italy), a centrifuge tube, and vortex mixer (Thermo Fisher Scientific, Rodano, Italy). Figure 2 provides a summary of the optimized DSLME method used for the extraction of phytochemicals from *C. sativa* L. aerial parts. In particular, 100 mg of the hemp sample was transferred to a centrifuge tube with 2 mL of KBr 30% aqueous solution and 100 μL of HDES. The mixture was then placed in a sonic bath (40 KHz at 25 °C) for 10 min after 30 s of vortexing. Once the extraction was complete, it was subjected to another 30 s of vortexing and centrifuged for 5 min at 4000 rpm. Three different phases were observed to form, starting from the bottom: (1) plant, (2) water, and (3) the HDES-rich phase. To allow easy isolation of the latter phase, it was resuspended in water, and the mixture (without the plant) was transferred in another tube and centrifuged again for 5 min at 4000 rpm. At this point, the aqueous phase was removed with a Pasteur pipette, and the remaining upper phase (HDES-rich phase) was diluted in 500 μL of MeOH/H_2_O (70:30, v/v). The extract was filtered with a 0.20 μm PVDF filter (CPS Analitica, Milan, Italy) prior to injection into the UHPLC-PDA system. The same procedure was used to simulate the extraction on 10 μg of pure standard compounds of luteolin-7-*O*-glucuronide, apigenin-7-*O*-glucuronide, and CBDA.

### UHPLC-PDA System and Operating Conditions

HPLC-grade acetonitrile was supplied by Merck Life Science S.r.l. (Milan, Italy). Deionized water (18.2 MΩ cm) was obtained from a Milli-Q purification system (Millipore, Bedford, MA, USA). Qualitative analyses were carried out with a Shimadzu UHPLC XR chromatograph equipped with a photodiode array detector SPD-M20A (Shimadzu, Dusseldorf, Germany) using an Ascentis Express C18 column (15 cm × 2.1 mm, 2.7 μm, Supelco, Bellefonte, USA). Separation of analytes was achieved using a binary mobile phase composed of water/formic acid (99.9:0.1, v/v) as phase A and acetonitrile/formic acid (99.9:0.1 v/v) as phase B. The gradient program is as follows: 0–2 min 15% B, 2–52 min 15–86% B, 52–55 min 86% B, and at a constant flow rate of 0.25 mL min^–1^. The total analysis time was 67 min. UV spectra were collected in the 220–450 nm wavelength range, and the resulting chromatograms were acquired the λ max of the identified peaks (270 nm for cannabinoid acids and 340 nm for flavonoid glycosides). Analyses were done in triplicate, and the analytical performance was measured in terms of repeatability. All HPLC data were processed using LabSolution software (Shimadzu, Dusseldorf Germany).

### Statistical Analysis

For IGC experiments, statistical calculations and multiple linear regression analysis were performed using Analyze-it (Microsoft, USA).

## Results and Discussion

### Synthesis of [Ch^+^][Br^–^]-Modified Salts

The aim of the first part of this study was to synthesize hydrophobic HBAs featuring a similar chemical structure to [Ch^+^][Cl^–^] through modification of its chemical structure. To obtain compounds with higher hydrophobicity, this study focused on the synthesis of choline derivatives with longer alkyl chain substituents while also maintaining the hydroxyl functional group. During preliminary tests, [Ch^+^][Br^–^] derivatives were found to be faster to synthesize and with a higher yield than the corresponding chlorine salts. Moreover, the bromide salts exhibited lower solubility in water (data not shown). Figure 1 shows the chemical structures of the three groups of salts prepared in this study. For synthesis of groups A and B, two different approaches were employed. Route (A) involved reaction of 2-dimethylaminoethanol and alkyl bromide to form surfactant-like salts featuring a more polar head and long alkyl chain substituents (Figure 1A) or route (B) where the amine and bromoethanol were reacted to form “bulky salts”, characterized by three carbon chain substituents of equal length as substituents of the ammonium group (Figure 1B). Tetrabutylammonium bromide and tetraoctylammonium bromide (Figure 1C) were chosen as representative commercial salts to compare their physicochemical properties with those of the synthesized [Ch^+^][Br^–^] derivative.

### Choice of the Most Suitable HBD for the Preparation of [Ch^+^][Br^–^]-Based HDES

After synthesizing HBAs, they were used in the preparation of HDESs. According to previous reports,^11−13^ several examples of alcohols, carboxylic acids, natural phenolics, and terpenoids have been employed as HBDs owing to their capability of interacting via hydrogen bonding. To reduce the number of candidates for testing, the main criteria used in choosing the HBD for combining with the synthesized [Ch^+^][Br^–^]-modified salts included the overall hydrophobicity, capability of forming a liquid HDES at room temperature, and cost. Moreover, compounds of natural origin enhance the greenness of the solvent. Ten compounds were tested as HBDs with different HBA/HBD ratios (see Table S5 of the Supporting Information). Thymol was able to easily form liquids with all six [Ch^+^][Br^–^]-modified salts at a molar ratio of 1:2 (see Table 1) and was therefore selected for subsequent studies.

### Evaluation of the Hydrophobicity of [Ch^+^][Br^–^]-Based HDES

**Figure 3:**
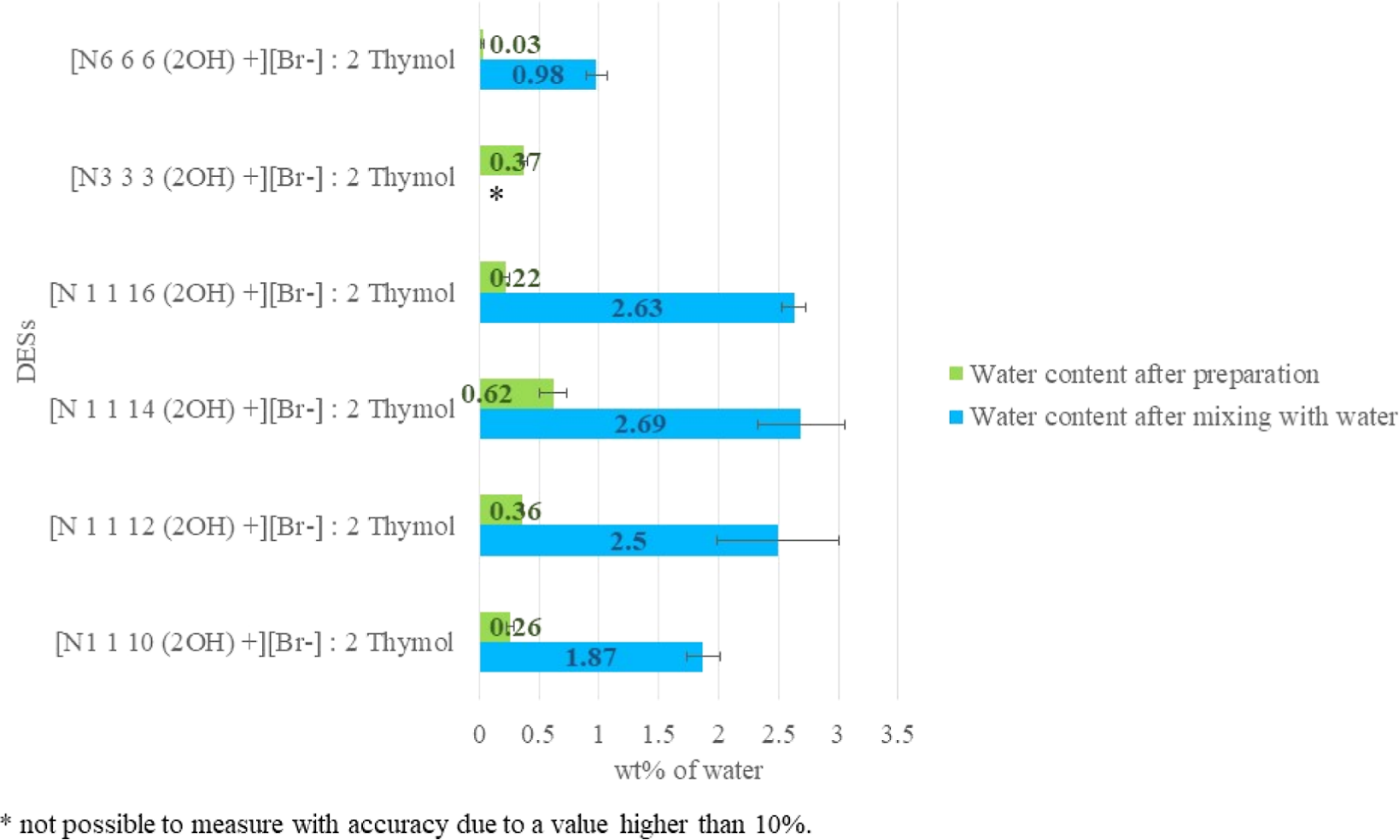
Water content (wt %) of [Ch^+^][Br^–^]-based HDESs measured by Karl Fischer titration, before and after mixing in water, expressed as the mean for *n* = 3 replicates.

Hydrophobicity of the proposed DESs is a fundamental parameter of this work to identify new hydrophobic solvents that can easily separate from water. Florindo and co-workers have reported^21^ that the hydrophobicity of DESs can be evaluated by measuring their miscibility in water. The initial water content of all the six synthesized salts was measured by Karl Fischer analysis after their preparation. The DESs were then placed in contact with water (see the Experimental Section for details) and the measurement was repeated. According to the literature,^13^ the initial water content of HDESs after preparation is reported to be in the range between 0.01 and 0.80 wt %, while when mixed with water, it can vary from 0.52 to 6.94 wt %. For [Ch^+^][Br^–^]-based HDESs, the water content after preparation varied from 0.03 to 0.62% wt and increased to 0.98–2.69 wt % after mixing with water, as shown in Figure 3. These results are in agreement with the reported values, proving the hydrophobic characteristic of the solvents. Only the [N_3 3 3 (2OH)_^+^][Br^–^] : 2Thymol DES yielded a water content higher than 10 wt % after mixing with water. Since the second part of this study involved the use of [Ch^+^][Br^–^]-based HDESs as extraction solvents in an aqueous solution, the stability of these eutectic mixtures in water was also monitored by ^1^H NMR. As reported in the Supporting Information, the [N_3 3 3 (2OH)_^+^][Br^–^] : 2 thymol DES was found to be partially soluble in water, revealing a higher hydrophilicity compared to the other [Ch^+^][Br^–^]-based HDESs. Despite the lower hydrophobicity of the [N_3 3 3 (2OH)_^+^][Br^–^] : 2 thymol DES, it was used in subsequent studies to compare its extraction efficiency with the other [Ch^+^][Br^–^]-based HDESs.

### Characterization of Selected [Ch^+^][Br^–^]-Based HDESs in Terms of Viscosity and Melting Point

The following four representative [Ch^+^][Br^–^]-based HDESs belonging to the three investigated groups of HDESs were selected for measurement of viscosity, melting point, and solvation properties: [N_1 1 10 (2OH)_^+^][Br^–^] : 2 thymol and [N_1 1 16 (2OH)_^+^][Br^–^] : 2 thymol for the surfactant-like salts, [N_3 3 3 (2OH)_^+^][Br^–^]/2 thymol from the bulky salts, and [N_4 4 4 4_^+^][Br^–^] : 2 thymol for the commercial salts. With regard to the thermal properties of the investigated HDESs, it can be observed from Table S2 that HDESs comprising surfactant-like HBAs often exhibited a melt peak, while no clear melting point could be identified for those comprising bulky salts. The viscosity measurements, reported in Table S3, showed that HDESs composed of bulky HBAs possessed significantly higher viscosities of up to 2228.33 cP ([N_4 4 4 4_^+^][Br^–^] : 2 thymol HDES) compared to only 138.96 cP for the [N_1 1 10 2(OH)_^+^][Br^–^] : 2 thymol HDES that comprised a surfactant-like salt.

### Characterizing Hydrophobic [Ch^+^][Br^–^]-Based HDESs Using Infrared Spectroscopy

To investigate the extent of hydrogen bonding between HBA and HBD, all HDESs were studied using ATR-FTIR spectroscopy. Figure S1 demonstrates the FTIR spectrum obtained for the [N_3 3 3 2(OH)_^+^][Br^–^] HBA. The vibrational band appearing at 3240 cm^–1^ corresponds to O–H stretching, while those occurring at 2969–2880 cm^–1^ refer to a C–H stretching of a sp^3^-hybridized functional group. In Figure S2, the FTIR spectrum for the thymol HBD is being shown. While the O–H stretching band was observed at 3283 cm^–1^, the C–H stretching of sp^2^ and sp^3^ alkyl substituents was obtained at 3067–3036 and 2958–2868 cm^–1^, respectively. Additionally, overtone bending was identified between 1875 and 1709 cm^–1^, and bands corresponding to C=C stretching are shown at 1621 cm^–1^. Figure S3 demonstrates the IR imaging for the [N_3 3 3 2(OH)_^+^][Br^–^] : 2 thymol DES, and bands related to functional groups of both HBA and HBD could be seen. It was observed that the O–H stretching band at 3263 cm^–1^ was significantly broader and much weaker compared to the corresponding band observed for HBA and HBD in Figures S1 and S2, indicating strong intermolecular hydrogen bonding interactions between the DES components in comparison to the starting materials. A similar phenomenon was noted for other HDESs compared to their components in Figures S4–S9, indicating that the hydroxyl functional groups of both HBA and HBD engage in strong hydrogen bonding interactions with the bromide hydrogen bond base in order to form stable HDESs.

### Influence of HBA on HDES Solvation Properties

Three independent methods were used to evaluate the solvation properties of hydrophobic [Ch^+^][Br^–^]-based HDESs. First, the most popular fluorescent dye polarity scales were employed. A detailed description of dyes, equations, and methods used to examine raw data from the solvatochromic dye method are provided in the Supporting Information. Given that only thymol was employed as the HBD and the HBA/HBD ratio was kept constant at 1:2 for all HDESs, only the effect of HBA was investigated. To estimate the polarity of bulky and surfactant-like HBAs, the Nile Red polarity scale (*E*_NR_) was employed. Table 2 provides the *E*_NR_ values for four HDESs examined in this study. The polarity values ranged from 50.21 ± 0.06 to 51.33 ± 0.05 and were generally very similar within the same class of HBA.

**Table 2:** *E*_NR_ Polarity Values and Kamlet–Taft Solvent Parameters for HDESs Composed of [N_1 1 10 2(OH)_^+^][Br^–^], [N_1 1 16 2(OH)_^+^][Br^–^], [N_3 3 3 2(OH)_^+^][Br^–^], and [N_4 4 4 4_^+^][Br^–^] HBAs and Thymol as the HBD^a^

HDES	*E*_NR_ (kcal/mol)	α	β	π*
[N_1 1 10 2(OH)_^+^][Br^–^] : 2 thymol	50.33 (0.05)	0.83 (0.02)	0.39 (0.02)	1.00 (0.03)
[N_1 1 16 2(OH)_^+^][Br^–^] : 2 thymol	50.45 (0.06)	0.80 (0.03)	0.33 (0.03)	0.98 (0.04)
[N_3 3 3 2(OH)_^+^][Br^–^] : 2 thymol	50.21 (0.06)	0.86 (0.03)	0.49 (0.01)	1.02 (0.02)
[N_4 4 4 4_^+^][Br^–^] : 2 thymol	51.33 (0.05)	0.66 (0.03)	0.60 (0.02)	0.93 (0.01)

a The *E*_NR_ values and the Kamlet–Taft solvent parameters are expressed as the mean for *n* = 3 replicates, and the standard deviation for each term is provided in parenthesis.

To better understand the types of solvation interactions, the same HDESs were further investigated using the Kamlet–Taft solvent parameters. Values for the hydrogen bond donating ability (α), hydrogen bond accepting ability (β), and dipolarity/polarizability (π*) are provided in Table 2. The hydrogen bond donating ability varied from 0.66 ± 0.03 to 0.86 ± 0.03 and was observed to be significantly higher for HDESs possessing a hydroxyl functional group in the HBA due to an additional acidic proton. Comparing the α-term values for the [N_1 1 10 2(OH)_^+^][Br^–^] : 2 thymol (0.83 ± 0.02), [N_1 1 16 2(OH)_^+^][Br^–^] : 2 thymol (0.80 ± 0.03), and [N_3 3 3 2(OH)_^+^][Br^–^] : 2 thymol HDESs (0.86 ± 0.03) reveals no significant difference in the hydrogen bond-donating ability between the bulky and surfactant-like hydrophobic [Ch^+^][Br^–^]-based HBAs. In contrast, β-term values were found to be 0.39 ± 0.02, 0.33 ± 0.03, and 0.49 ± 0.01 for the [N_1 1 10 2(OH)_^+^][Br^–^] : 2 thymol, [N_1 1 16 2(OH)_^+^][Br^–^] : 2 thymol, and [N_3 3 3 2(OH)_^+^][Br^–^] : 2 thymol HDESs, respectively, indicating that the hydrogen bond accepting ability varies significantly between bulky and surfactant-like HBAs as well as within the same class. The [N_3 3 3 2(OH)_^+^][Br^–^] : 2 thymol DES possessed a drastically lower hydrogen bond accepting ability compared to the [N_4 4 4 4_^+^][Br^–^] : 2 thymol HDES (0.60 ± 0.02), which can be attributed to the presence of an additional acidic proton on the hydroxyl functional group that can interact with the bromide anion and reduce its hydrogen bond basicity. In addition, HDESs comprising bulky HBAs possessed higher hydrogen bond accepting ability compared to those comprising surfactant-like salts, irrespective of the presence/absence of the hydroxyl functional group. Moreover, dipolar interactions were found to range from 0.93 ± 0.01 to 1.02 ± 0.02, but no specific trend could be determined for the two types of HBAs employed in this study.

HDESs comprising [N_1 1 10 2(OH)_^+^][Br^–^], [N_1 1 16 2(OH)_^+^][Br^–^], [N_3 3 3 2(OH)_^+^][Br^–^], [N_4 4 4 4_^+^][Br^–^], and [N_6 6 6 2(OH)_^+^][Br^–^] salts as the HBA were further examined using IGC between 40 and 60 °C. Given the volatile nature of thymol, triethylene glycol (TEG) was chosen as the HBD in the preparation of DESs for IGC studies. All salts formed a homogeneous eutectic mixture with TEG at a molar ratio of 1:4; this molar ratio was kept constant in order to investigate the effect of HBA on DES solvation interactions. The unique solvation interactions were deconvoluted using the Abraham solvation parameter model, and a detailed description of equations and methods used to generate system constants from the model is provided in the Supporting Information. Figure S10 exhibits a representative regression line consisting of 35 probe molecules with a correlation coefficient (*R*) value of 0.99. As shown in Table 3, all models generated in this study possessed a high magnitude of Fisher *F*-statistics and correlation coefficients (0.99) of the multiple linear regression fit. Dipolar interactions were generally found to vary between 1.79 ± 0.07 and 2.25 ± 0.06 at 60 °C and were observed to be lower for DESs comprising surfactant-like HBAs compared to those composed of bulky salts. The strength of dispersive-type interactions was generally dependent on the length of alkyl substituents on the cation with the lowest value of 0.52 ± 0.01 obtained for the [N_3 3 3 2(OH)_^+^][Br^–^] : 4 TEG DES and the highest *l*-term value of 0.67 ± 0.01 exhibited by the [N_1 1 16 2(OH)_^+^][Br^–^] : 4 TEG DES. DESs comprising bulky salts generally offered lower dispersive-type interactions compared to those comprising surfactant-like HBAs.

**Table 3:** System Constants for DESs Comprising [N_1 1 10 2(OH)_^+^][Br^–^], [N_1 1 16 2(OH)_^+^][Br^–^], [N_3 3 3 2(OH)_^+^][Br^–^], and [N_4 4 4 4_^+^][Br^–^] HBAs and TEG as the HBD^a^

		system constants								
DES^a^	temp. (°C)	*c*	*e*	*s*	*a*	*b*	*l*	*n*^b^	*R*^2^^c^	*F*^d^
	40	–3.45 (0.05)	0 (0)	2.20 (0.07)	4.66 (0.10)	0.11 (0.09)	0.70 (0.01)	35	0.99	1751
[N_1 1 10 2(OH)_^+^][Br^–^] : 4TEG	50	–3.32 (0.05)	0.07 (0.05)	2.11 (0.06)	4.29 (0.09)	0 (0)	0.63 (0.01)	35	0.99	1543
	60	–3.31 (0.05)	0 (0)	2.07 (0.06)	4.10 (0.09)	0 (0)	0.60 (0.01)	35	0.99	1567
	40	–3.43 (0.10)	–0.13 (0.10)	1.86 (0.12)	4.26 (0.11)	0 (0)	0.83 (0.02)	35	0.99	942
[N_1 1 16 2(OH)_^+^][Br^–^] : 4TEG	50	–3.56 (0.09)	–0.17 (0.10)	1.75 (0.12)	4.03 (0.10)	0 (0)	0.80 (0.02)	35	0.99	1038
	60	–3.21 (0.05)	0 (0)	1.79 (0.07)	3.76 (0.11)	0 (0)	0.67 (0.01)	35	0.99	1168
[N_3 3 3 2(OH)_^+^][Br^–^] : 4TEG	40	–3.15 (0.05)	0.16 (0.06)	2.29 (0.07)	4.40 (0.11)	0.23 (0.09)	0.59 (0.01)	35	0.99	1278
	50	–3.14 (0.06)	0.16 (0.06)	2.17 (0.07)	4.08 (0.11)	0.20 (0.09)	0.55 (0.01)	35	0.99	1079
	60	–3.20 (0.05)	0.14 (0.05)	2.18 (0.06)	4.02 (0.10)	0.10 (0.08)	0.52 (0.01)	35	0.99	1225
[N_4 4 4 4_^+^][Br^–^] : 4TEG	40	–3.23 (0.05)	0 (0)	2.40 (0.07)	4.79 (0.11)	0 (0)	0.65 (0.01)	35	0.99	1377
	50	–3.21 (0.05)	0 (0)	2.31 (0.06)	4.46 (0.10)	0 (0)	0.61 (0.01)	35	0.99	1423
	60	–3.25 (0.05)	0 (0)	2.25 (0.06)	4.22 (0.10)	0 (0)	0.58 (0.01)	35	0.99	1389
[N_6 6 6 2(OH)_^+^][Br^–^] : 4TEG	40	–3.19 (0.09)	–0.02 (0.07)	2.28 (0.08)	4.58 (0.15)	–0.15 (0.10)	0.78 (0.01)	35	0.99	610
	50	–3.23 (0.08)	–0.01 (0.07)	2.24 (0.09)	4.35 (0.14)	–0.12 (0.09)	0.73 (0.01)	35	0.99	572
	60	–3.37 (0.07)	–0.07 (0.07)	2.15 (0.09)	4.28 (0.14)	–0.13 (0.12)	0.64 (0.01)	35	0.99	638

a The chemical structures and molecular weights of HBAs and HBDs can be found in Figure 1.

b *n*, number of probes subjected to multiple linear regression analysis.

c *R*^2^, correlation coefficient.

d *F*, Fisher *F*-statistic.

Like most classes of DESs, hydrogen bonding was among the strongest type of solvation interaction measured. The hydrogen bond basicity ranged from 3.76 ± 0.11 to 4.28 ± 0.14 at 60 °C and was often observed to be higher for DESs comprising bulky salts compared to surfactant-like HBAs. Within the class of bulky HBAs, DESs that possessed longer alkyl substituents in the cation exhibited higher hydrogen bond basicity, which is consistent with the trend previously reported for both tetraalkylammonium and [Ch^+^][Cl^–^]-based DESs.^22,23^ In contrast, longer alkyl functional groups in surfactant-like HBAs resulted in lower hydrogen bond basicity, as observed in the *b*-term values for the [N_1 1 10 2(OH)_^+^][Br^–^] : 4 TEG (4.10 ± 0.09) and [N_1 1 16 2(OH)_^+^][Br^–^] : 4 TEG (3.76 ± 0.11) DESs at 60 °C. These trends are further supported by the chromatographic retention behavior of alcohols (provided in Table S6), which are acidic in nature and can strongly interact with the bromide anion. Additionally, the retention characteristics of basic probe molecules, such as *N*,*N*-DMAC, also provide insights into the relative strength of hydrogen bond acidity in these DESs. It was observed that *N*,*N*-DMAC produced retention factors as high as 48.04 (Table S6) on the [N_6 6 6 2(OH)_^+^][Br^–^]/4 TEG DES compared to only 37.04 on the [N_1 1 16 2(OH)_^+^][Br^–^] : 4 TEG DES at 60 °C, indicating that bulky salts exhibit higher hydrogen bond acidity compared to surfactant-like HBAs.

Despite both being totally different approaches, it is vital that the results of the Kamlet–Taft solvent parameters and the Abraham solvation parameter model are in complete agreement with each other for solvation interactions that can be quantified using both methods, such as hydrogen bond basicity and dipolarity. The trend in relative strength for hydrogen bond accepting ability (β) is observed to be as follows: [N_1 1 16 2(OH)_^+^][Br^–^] < [N_1 1 10 2(OH)_^+^][Br^–^] < [N_3 3 3 2(OH)_^+^][Br^–^] < [N_4 4 4 4_^+^][Br^–^]. Comparing this with the *a*-term for the same DESs revealed a similar trend for the same set of DESs. Clearly, both methods indicate that surfactant-like HBAs offer lower hydrogen bond basicity compared to bulky salts. This is useful in explaining some trends observed in the extraction of flavonoids and cannabinoids. It was observed that flavonoids, such as luteolin-7-*O*-glucuronide and apigenin-7-*O*-glucuronide, generally resulted in higher extraction with DESs that exhibit lower hydrogen bond basicity.

### [Ch^+^][Br^–^]-Based HDES for the Extraction of Phytochemicals with Different Polarities from *C. sativa* L. Samples

After characterization of the [Ch^+^][Br^–^]-based HDESs, their applicability in the extraction of natural compounds was explored. Apart from the extraction performance of the new solvents, their compatibility with liquid chromatography was also investigated since chromatographic techniques are fundamental for the analysis and study of plant metabolome to separate and identify the analytes of interest in complex samples. In this sense, it was important to verify that the signal of thymol (HBD in the HDESs) in the chromatogram did not interfere with the signal of the target analytes. Moreover, blank injections of MeOH 100% were regularly repeated within the analysis of [Ch^+^][Br^–^]-based HDES extracts to detect potential carryover.

When dealing with plant matrices, solvents able to extract a wide range of phytochemicals are fundamental in obtaining extracts representative of the plant metabolome. The simultaneous presence of hydrophilic domains (hydroxyl group and charge of ammonium) and long alkyl chain substituents in the structure of [Ch^+^][Br^–^]-based HDESs may promote the extraction of phytochemicals with different polarities. The aerial parts of fiber-type *C. sativa* L. were chosen due to the rich phytocomplex comprising several classes of non-volatile bioactive compounds. As reported by the authors,^24^ the main phytochemicals identified in this matrix were flavonoid glycosides and non-psychotomimetic cannabinoid acids. Previous studies on neutral HDESs composed of terpenoids compounds of natural origin have highlighted that these solvents were capable of extracting cannabinoid compounds but less effective in the enrichment of the flavonoid fraction.^16^

### Optimization of the DSLME Method for the Analysis of *C. sativa* L. Aerial Parts

**Figure 4:**
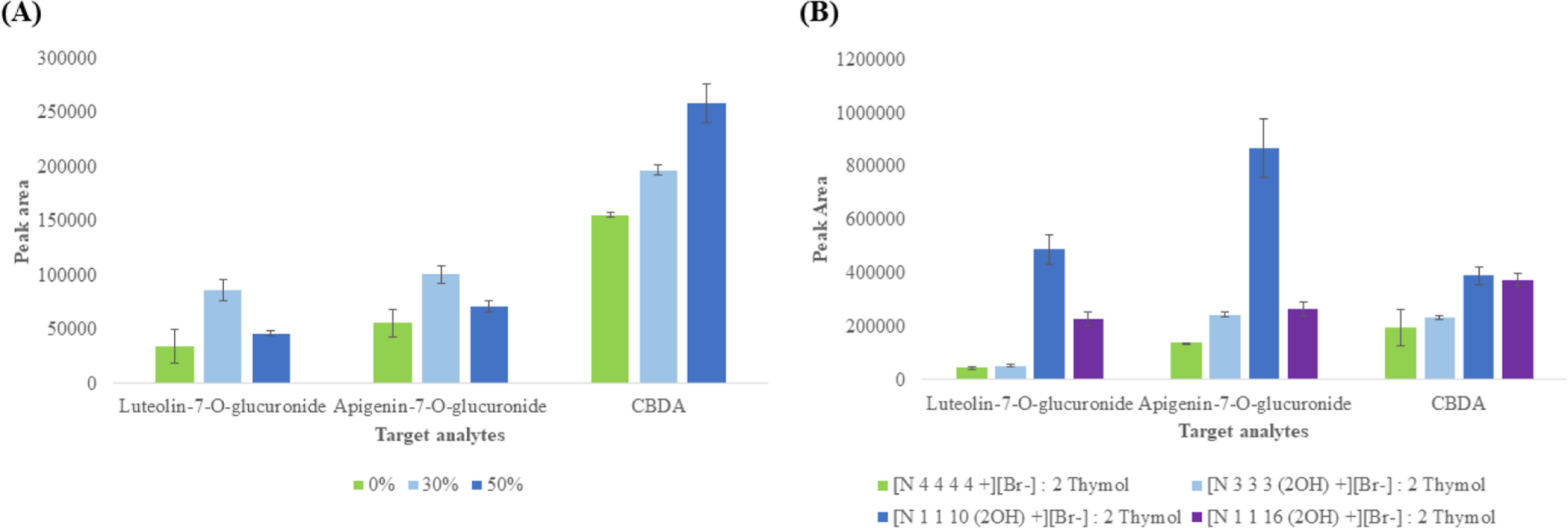
(A) Optimization of KBr content (% of saturation concentration) in the DSLME method using the [N_1 1 16 (2OH)_^+^][Br^–^] : 2 thymol HDES as a solvent (*n* = 3) and (B) evaluation of the extraction performance (evaluated as peak area) of the HDESs under study for the three target analytes/luteolin-7-*O*-glucuronide, apigenin-7-*O*-glucuronide, and CBDA. The results are expressed as the mean for *n* = 3 replicates.

To evaluate the extraction performance of the proposed HDESs for all target analytes identified in the hemp sample, a DSLME method was first applied to a mix of representative standard compounds (e.g., luteolin-7-*O*-glucuronide, apigenin-7-*O*-glucuronide, and CBDA) in an effort to simulate their behavior in a less complex system. The DSLME approach was employed for simplicity and speed of the procedure while also reducing the amount of solvent to improve the enrichment of analytes in the final HDES extract. To reduce the number of experiments, the ([N_1 1 16 (2OH)_^+^][Br^–^] : 2 thymol) HDES was selected for use in the first part of the optimization. The extraction was performed on 10 μg of the three standards following the procedure reported in Figure 2. To improve transfer of analytes from the water layer to the HDES, the addition of aqueous solutions of KBr (30% (w/w) and 50% (w/w)) of the saturation concentration) was also tested. Although NaCl is usually employed,^25^ KBr was used to prevent an ion-exchange reaction between the [Cl^–^] anion of NaCl and the [Br^–^] anion of [Ch^+^][Br^–^]-based HDESs during the extraction step. Figure 4A shows the results obtained when 0% (w/w), 30% (w/w), and 50% (w/w) of KBr salt were examined. As expected, the addition of 30% KBr decreased the solubility in water of the three analytes, thus favoring their partitioning to the HDES phase. A further increase of KBr in the water solution (50%) improved the enrichment of CBDA, while the extraction efficiency of flavonoid glycosides was lower compared to that observed at 30% KBr. These results are in agreement with previous studies in which a lower extraction efficiency at very high salt concentration was reported.^26−28^ This behavior can be ascribed to the increase of viscosity of the aqueous solution, which results in a slower mass transfer. For this reason, an aqueous solution containing 30% of KBr was selected as an optimal solvent for the following steps.

Once the method was optimized, it was applied to study the extraction performance of the [Ch^+^][Br^–^]-based HDESs on pure standard compounds. Figure 4B shows the extraction efficiencies for the three analytes, expressed as chromatographic peak areas. In general, HDESs formed from surfactant-like salts exhibit higher extraction performance for all compounds, with a clear predominance of the [N_1 1 10 (2OH)_^+^][Br^–^] : 2 thymol HDES in the extraction of flavonoid glycosides, while this difference is less marked for CBDA. It is noteworthy that the lower peak area of flavonoid glycosides, observed in the [N_1 1 16 (2OH)_^+^][Br^–^] : 2 thymol extract, may be due to the breakage of bonds between the aglycone (luteolin or apigenin) and the sugar (glucuronic acid) during extraction. To support this, Figure S11 of the Supporting Information shows the presence of two additional peaks in the chromatographic profile of the extract, which were identified as luteolin and apigenin aglycone. These peaks are not present in the other extracts, suggesting that the long alkyl chain substituents within the [N_1 1 16 (2OH)_^+^][Br^–^] salts may undergo interaction with the glycosidic bond present in the flavonoid glycosides.

**Figure 5:**
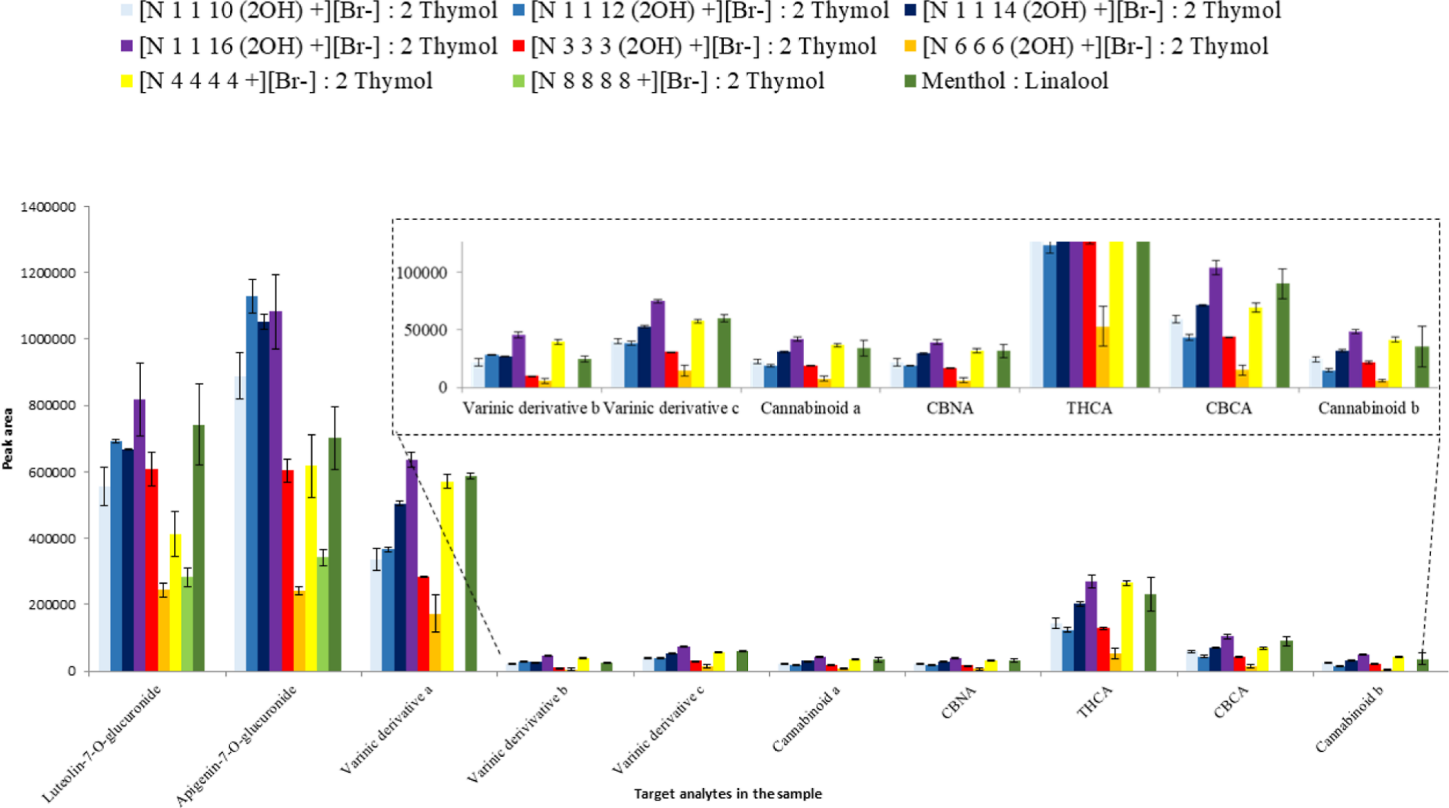
Extraction performance (evaluated as peak area and expressed as the mean for *n* = 3 replicates) of the investigated HDESs in the analysis of the non-volatile fraction of fiber-type *C. sativa* L. aerial parts. The identification of the analytes follows the one reported in by the authors.^23^ Cannabinolic acid, tetrahydrocannabinolic acid, cannabichromenic acid, varinic derivative A/B, and cannabinoid A/B were putatively identified at class level through retention times, maximum UV adsorption, and tandem mass spectrometry fragmentation patterns.

Once the method was optimized for standard compounds, it was applied for the extraction of the non-volatile fraction of fiber-type *C. sativa* L. aerial parts (see Figure 2). In this case, the extraction was carried out with all the six [Ch^+^][Br^–^]-based HDESs to investigate differences in the extraction performance between all solvents. For comparison purposes, the optimized DSLME method was also performed with the two HDESs formed by the commercial ammonium salts and with a eutectic mixture composed of menthol and linalool. The menthol/linalool mixture was selected as a reference because it was previously employed as an extraction solvent for the same hemp sample.^16^Figure 5 shows the extraction efficiencies obtained for the target compounds expressed as chromatographic peak areas. The behavior of the [Ch^+^][Br^–^]-based HDES, when the method was applied on the plant, slightly differs from that observed in the aqueous solution (see the previous paragraph).

With regard to the flavonoid fraction, the peak areas were observed to increase in accordance with an increase of the apolar tail length (from C10 to C16) of the surfactant-like salts. In this case, the aglycones are not detected, indicating that the plant matrix prevents degradation of the glycosidic bond. This may explain why, contrary to extraction from aqueous solution, the [N_1 1 16 (2OH)_^+^][Br^–^] : 2 thymol HDES was more effective than the [N_1 1 10 (2OH)_^+^][Br^–^]/2 thymol HDES in terms of extraction performance. As previously observed in the extraction of pure compounds, the two HDESs based on [Ch^+^][Br^–^]-modified bulky salts provide lower efficiency, with higher peak areas obtained for the [N_3 3 3 (2OH)_^+^][Br^–^] : 2 thymol HDES. A similar trend is observed for the HDESs based on commercial salts, where the shorter chains of the [N_4 4 4 4_^+^][Br^–^]/2 thymol HDES appear to extract more analytes compared to the [N_8 8 8 8_^+^][Br^–^] : 2 thymol HDES. The peculiar chemical structure of the surfactant-like salts characterized by a long alkyl chain substituent may assist in the interaction of the solvent with the plant’s cell wall, which is necessary for the release of the target compounds. On the other hand, larger steric hindrance around the ammonium group in the other salts may negatively affect the interaction with the analytes. Moreover, the viscosity data obtained for selected HDESs (see Table S3) shows that bulky and commercial salts provide higher viscosity compared to surfactant-like HDESs. This suggests that the lower extraction efficiency of HDESs based on bulky and commercial salts could also be a result of their higher viscosity, which may interfere with transfer of the analytes from the plant to the solvent.^29^ This is confirmed by the extraction efficiency offered by the menthol/linalool eutectic mixture, which is higher or similar to the bulky/commercial salt-based HDESs. Despite the higher hydrophobicity of the terpenoid mixture, the viscosity of neutral HDESs is typically much lower compared to that of the ionic ones.^30^

Regarding cannabinoid compounds, the extraction efficiency of the different HDESs is similar to those observed for flavonoids, except for the [N_4 4 4 4_^+^][Br^–^] : 2 thymol HDES, which follows a similar trend to that of the [N_1 1 16 (2OH)_^+^][Br^–^] : 2 thymol HDES. This could be justified by the lower polarity of the [N_4 4 4 4_^+^][Br^–^] : 2 thymol HDES, which provides more favorable interactions with cannabinoids compared to the more polar flavonoid glycosides. The high viscosity of the [N_8 8 8 8_^+^][Br^–^] : 2 thymol HDES negatively affected extraction of the cannabinoid fraction, whose peaks were not detected in the chromatographic profile.

Based on these results, the [N_1 1 16 (2OH)_^+^][Br^–^]/2 thymol HDES appeared to be the best among all eutectic mixtures tested with regard to analysis of phytochemicals with different polarities in *C. sativa* L. aerial parts. It exhibited higher enrichment of more polar compounds while maintaining a similar extraction efficiency of cannabinoids compared to the reference menthol/linalool hydrophobic solvent. It is also interesting to highlight that [N_1 1 16 (2OH)_^+^][Br^–^] and surfactant-like salts, in general, consist of simpler and faster synthetic processes and higher yields compared to the bulky salts, thus enabling a more reliable application in analytical procedures.

## Conclusions

One of the main features and advantages of DESs is the possibility to easily vary their physicochemical properties by choosing the adequate HBAs and HBDs. Hydrophilic DESs are the most studied due to the possibility of using a wide range of hydrophilic and cheap compounds able to interact through hydrogen bonding. Because hydrophilic DESs are unstable in aqueous solution, their applicability is limited, and hydrophobic DESs are rapidly emerging. However, it is more challenging to find hydrophobic compounds that easily form a hydrogen bonding network for the preparation of HDESs. [Ch^+^][Cl^–^] has been widely used in the preparation of hydrophilic eutectic mixtures due to its capacity of interaction with many different HBDs. For this reason, the chemical structure of choline was used as a model to synthesize a new class of hydrophobic HBAs. Six different hydrophobic [Ch^+^][Br^–^] derivatives were synthesized by increasing the length of alkyl chain substituents while maintaining the hydroxyl functional group. The [Ch^+^][Br^–^]-modified salts were mixed with thymol to obtain HDESs, which were then characterized in terms of hydrophobicity, viscosity, and solvation properties. To test their applicability in extraction studies, [Ch^+^][Br^–^]-based HDESs were employed as solvents in a DSLME method for the analysis of *C. sativa* L. plant, characterized by a unique phytocomplex that includes compounds with different polarities. In this regard, the hydrophobic features of [Ch^+^][Br^–^]-based HDESs, together with hydrophilic domains (hydroxyl group and charge on the ammonium group) in their structure, facilitated the interaction with both polar and less polar compounds. In general, the lower viscosity of the [Ch^+^][Br^–^]-based HDESs, compared to eutectic mixtures based on conventional hydrophobic HBAs, promoted the extraction of flavonoids and cannabinoids. Moreover, the extraction of flavonoids appeared to be enhanced by [Ch^+^][Br^–^]-based HDESs with lower hydrogen bond basicity. Among the [Ch^+^][Br^–^]-based DESs tested, the HDES comprising the [N_1 1 16 (2OH)_^+^][Br^–^] salt demonstrated higher extraction capacity due to its low viscosity and peculiar solvation properties. Due to the promising extraction performances of the [Ch^+^][Br^–^]-based HDESs for compounds of varying polarity in *C. sativa*, future advances will be directed to the application and validation of the DSLME method to different *C. sativa* samples.

## Supporting Information Available

Solvatochromic dye methods and Abraham solvation parameter model, stability of HDESs during extraction, reaction conditions for ChBr-modified salts under study, HBDs and molar ratio tested to form HDESs with ChBr-modified salts, ATR–FTIR spectra, multiple linear regression plot for the [N_1 1 10 2(OH)_^+^][Br^–^]/4 TEG DES, chromatographic profile of the DSLME of standard compounds, and NMR spectra (PDF)
