# Supplementary material for: New Class of Tunable Choline Bromide-Based Hydrophobic Deep Eutectic Solvents for the Extraction of Bioactive Compounds of Varying Polarity from a Plant Matrix
Source: ACS Sustain Chem Eng. 2023 Apr 20;11(17):6665–75. doi: 10.1021/acssuschemeng.3c00185 (PMC10155672; doi:10.1021/acssuschemeng.3c00185)
Supplement: Supplementary file 1 — sc3c00185_si_001.pdf [file sc3c00185_si_001.pdf]

Supporting Information

New class of tunable choline bromide-based hydrophobic deep eutectic solvents for the extraction of bioactive compounds of varying polarity from a plant matrix

Giulia Mastellone[†$], Nabeel Mujtaba Abbasi[‡$], Cecilia Cagliero [†,*] and
Jared L. Anderson[‡]

[†] *Department of Drug Science and Technology, University of Turin, Via Pietro Giuria, 9,I-10125 Torino, Italy*
[‡] *Ames National Laboratory—USDOE and Department of Chemistry, Iowa State University, 1605 Gilman Hall, Ames, Iowa 50011, USA*

Supporting information contents:

Number of pages: 41

Number of figures: 36

Number of tables: 6

INDEX

Solvatochromic dye methods……………………………………………………Page S3

The Abraham solvation parameter model ………………………………………Page S4

Stability of HDESs during the extraction ………………………………………Page S5

Table S1……………………………………………………………………Page S6

Table S2……………………………………………………………………Page S6

Table S3……………………………………………………………………Page S6

Table S4……………………………………………………………………Pages S7-S8

Table S5……………………………………………………………………Page S8

Table S6……………………………………………………………………Page S9

Figure S1…………………………………………………………………...Page S10

Figure S2…………………………………………………………………...Page S10

Figure S3…………………………………………………………………...Page S11

Figure S4…………………………………………………………………...Page S11

Figure S5…………………………………………………………………...Page S12

Figure S6…………………………………………………………………...Page S12

Figure S7…………………………………………………………………...Page S13

Figure S8…………………………………………………………………...Page S13

Figure S9…………………………………………………………………...Page S14

Figure S10…………………………………………………………………Page S15

Figure S11…………………………………………………………………Page S16

NMR Spectra (figures S12-S36)...…………………………………………Pages S17-S40

References..………………………………………………………………...Page S41

Solvatochromic dye methods

The choice of dyes employed in calculating Kamlet-Taft solvent parameters are based on a previous approach.[1] Equations 1-4 were used to determine the (E_{NR}) values and Kamlet-Taft solvent parameters:

$$E_{NR}\ (\text{kcal/mol}) = 28591 / \Lambda_{max}\ (\text{nm})\ (1)$$

$$\alpha = (19.9657 - 1.0241\pi^* - \upsilon_{NR}) / 1.6078\ (2)$$

$$\beta = (1.035\ \upsilon_{DENA} + 2.64 - \upsilon_{4\text{-}NA}) / 2.80\ (3)$$

$$\pi^* = 0.314\ (27.52 - \upsilon_{DENA})\ (4)$$

$$\text{where } \upsilon = 1 / (\Lambda_{max} \times 10^{-4})$$

The Abraham solvation parameter model

To relate chromatographic retention of analytes to individual solute-solvent interactions, the Abraham solvation parameter model (equation 5) was employed.

$$Log\ k = c + eE + sS + aA + bB + lL \quad (5)$$

In equation 5, *k* represents the retention factor of each probe molecule calculated using the dead time and the retention time of each chromatographic column. The solute descriptors (*E, S, A, B,* and *L*) are specific to analytes and represent the excess molar refraction, dipolarity/polarizability, hydrogen bond acidity, hydrogen bond basicity, and gas-hexadecane partition coefficient at 298 K, respectively. The system constants (*e*, *s*, *a*, *b*, and *l*) represent specific solvation interactions of the DES stationary phase, where *e* measures n-π and π-π interactions, *s* the dipolarity/polarizability, *a* the hydrogen bond basicity, *b* the hydrogen bond acidity, and *l* provides a measure of dispersive-type interactions.

Stability of HDESs during the extraction

Due to its capacity in undergoing hydrogen bonding, water is supposed to undergo interaction with the hydrogen bonding network of DESs. [2] For this reason, it is important to verify their stability when performing applications involving aqueous solutions. In the DSLME method, an aqueous solution of 30% KBr is used as co-solvent to form a dispersion of the HDES in the sample. To verify that the HDES remains intact during extraction, its ^1H NMR profile was compared before and after extraction. To avoid interference of the plant and analytes in the NMR spectra, the extraction was carried out by substituting the plant material with 2 mL of KBr aqueous solution. The extraction was then carried out as reported in Figure 2 and the recovered HDESs diluted in deuterated solvent and subjected to NMR measurements. For all HDESs, ^1H NMR spectra showed the presence of both the HBA and HBD after the extraction, confirming that hydrogen bonding is intact after dispersion in water and that the entire HDES is responsible for the extraction. The molar ratio between the HBA and HBD was also monitored and showed to be stable after extraction. The only exception was the [$N_{3\,3\,3\,(2OH)}^{+}$] : 2 Thymol HDES, where the molar ratio between [$N_{3\,3\,3\,(2OH)}^{+}$] and Thymol changed after the extraction process. In fact, part of [$N_{3\,3\,3\,(2OH)}^{+}$] dissolved in the water phase during the extraction and it was therefore found in lower relative amounts in the HDES phase. This behavior appears to be linked to its higher hydrophilicity, already observed in the measurement of water content. The ^1H NMR spectra of [$N_{3\,3\,3\,(2OH)}^{+}$] : 2Thymol and [$N_{1\,1\,16\,(2OH)}^{+}$] : 2 Thymol before and after the extraction are shown as representative examples in the supporting information.

Table S1. Reaction conditions of the $[Ch^+][Br^-]$-modified salts synthesized in the study.

ChBr-modified salts	Reagent 1	Reagent 2	Solvent	Molar ratio	Purification	Yield (%)	Characterization	Ref.
$[N_{1\,1\,10\,(2OH)}{}^+][Br^-]$	Dimethylammino ethanol	1-Bromodecane	Hexane	1 : 1.1	Wash with hexane	50%	^1H and ^{13}C NMR	18
$[N_{1\,1\,12\,(2OH)}{}^+][Br^-]$	Dimethylammino ethanol	1-Bromododecane	Hexane	1 : 1.1	Wash with hexane	70%	^1H and ^{13}C NMR	18
$[N_{1\,1\,14\,(2OH)}{}^+][Br^-]$	Dimethylammino ethanol	1-Bromotetradecane	Hexane	1 : 1.1	Wash with hexane	70%	^1H and ^{13}C NMR	18
$[N_{1\,1\,16\,(2OH)}{}^+][Br^-]$	Dimethylammino ethanol	1-Bromohexadecane	Hexane	1 : 1.1	Wash with hexane	70%	^1H and ^{13}C NMR	18
$[N_{3\,3\,3\,(2OH)}{}^+][Br^-]$	Tripropylamine	Bromoethanol	Toluene	1 : 1	Rotary evaporation + wash with diethyl ether	30%	^1H and ^{13}C NMR	17
$[N_{6\,6\,6\,(2OH)}{}^+][Br^-]$	Trihexylamine	Bromoethanol	Toluene	1.2 : 1	Rotary evaporation, wash with diethyl ether + crystallization in ethyl acetate	30%	^1H and ^{13}C NMR	17

Table S2. Melting peak/glass transition temperatures of 25 HDESs as measured by a Thermal Advantage DSC Q2000 instrument.

HDES	T_g (℃)	T_m (℃)
$[N_{1\,1\,10\,2(OH)}{}^+][Br^-]$: 2 Thymol	-64.2	-
$[N_{1\,1\,16\,2(OH)}{}^+][Br^-]$: 2 Thymol	-84.0	6.9
$[N_{3\,3\,3\,2(OH)}{}^+][Br^-]$: 2 Thymol	-44.1	-
$[N_{4\,4\,4\,4}{}^+][Br^-]$: 2 Thymol	-43.2	-
$[N_{6\,6\,6\,2(OH)}{}^+][Br^-]$: 2 Thymol	-89.9	-

"-" No T_m/T_g peak was observed/reported

Table S3. Viscosity data obtained at 21.6 °C for all HDESs.

HDES/neat HBDs	Viscosity (cP)
$[N_{1\,1\,10\,2(OH)}{}^+][Br^-]$: 2 Thymol	138.96
$[N_{1\,1\,16\,2(OH)}{}^+][Br^-]$: 2 Thymol	96.14
$[N_{3\,3\,3\,2(OH)}{}^+][Br^-]$: 2 Thymol	1856.33
$[N_{4\,4\,4\,4}{}^+][Br^-]$: 2 Thymol	2228.33
$[N_{1\,1\,1\,2(OH)}{}^+][Br^-]$: 2 Thymol ($[Ch^+][Br^-]$) : 2 Thymol	-[a]

[a] HDES resembled a chunky solid at 21.6 °C

Table S4. List of all probe molecules and their corresponding solute descriptors employed in this study.

Probe Molecule	E	S	A	B	L
Acetophenone	0.818	1.01	0	0.48	4.501
Benzaldehyde	0.82	1	0	0.39	4.008
Benzene	0.61	0.52	0	0.14	2.786
Benzonitrile	0.742	1.11	0	0.33	4.039
Bromoethane	0.366	0.4	0	0.12	2.62
1-Bromooctane	0.339	0.4	0	0.12	5.09
1-Butanol	0.224	0.42	0.37	0.48	2.601
1-Chlorobutane	0.21	0.4	0	0.1	2.722
1-Chlorohexane	0.201	0.4	0	0.1	3.777
1-Chlorooctane	0.191	0.4	0	0.1	4.772
Cyclohexanol	0.46	0.54	0.32	0.57	3.758
Cyclohexanone	0.403	0.86	0	0.56	3.792
1,2-Dichlorobenzene	0.872	0.78	0	0.04	4.518
N,N-Dimethylformamide	0.367	1.31	0	0.74	3.173
1,4-Dioxane	0.329	0.75	0	0.64	2.892
Ethyl Acetate	0.106	0.62	0	0.45	2.314
Ethyl benzene	0.613	0.51	0	0.15	3.778
1-Iodobutane	0.628	0.4	0	0.15	4.13
Methyl Caproate	0.067	0.6	0	0.45	3.844
Naphthalene	1.34	0.92	0	0.2	5.161
Nitrobenzene	0.871	1.11	0	0.28	4.557
1-Nitropropane	0.242	0.95	0	0.31	2.894
Octylaldehyde	0.16	0.65	0	0.45	4.361
1-Pentanol	0.219	0.42	0.37	0.48	3.106
2-Pentanone	0.143	0.68	0	0.51	2.755
Ethyl phenyl ether	0.681	0.7	0	0.32	4.242
Propionitrile	0.162	0.9	0.02	0.36	2.082
Pyridine	0.631	0.84	0	0.52	3.022
Pyrrole	0.613	0.73	0.41	0.29	2.865
Toluene	0.601	0.52	0	0.14	3.325
m-Xylene	0.623	0.52	0	0.16	3.839
o-Xylene	0.663	0.56	0	0.16	3.939
p-Xylene	0.613	0.52	0	0.16	3.839
2-Propanol	0.212	0.36	0.33	0.56	1.764
2-Nitrophenol	1.015	1.05	0.05	0.37	4.76
1-Bromohexane	0.349	0.4	0	0.12	4.13
1-Decanol	0.191	0.42	0.37	0.48	5.628
Methanol	0.278	0.44	0.43	0.47	0.97
Ethanol	0.246	0.42	0.37	0.48	1.485
Nitromethane	0.313	0.95	0.06	0.31	1.892
Cyclopentanol	0.427	0.54	0.32	0.56	3.241
Cycloheptanol	0.513	0.54	0.32	0.58	4.407
Phenylethyne	0.679	0.58	0.12	0.24	3.692

1-Hexanol	0.21	0.42	0.37	0.48	3.61
Bromobutane	0.36	0.4	0	0.12	3.105
Iodoethane	0.64	0.4	0	0.15	2.573
Methylacetate	0.142	0.64	0	0.45	1.911
2-Butanol	0.217	0.36	0.33	0.56	2.338
1-Propanol	0.236	0.42	0.37	0.48	2.031
Pyrazine	0.629	0.95	0	0.62	2.920
2-Methylpyrazine	0.629	0.90	0	0.64	3.254
N,N-Dimethylacetamide	0.363	1.33	0	0.78	3.717

Table S5. Combinations and molar ratio of $[Ch^{+}][Br^{-}]$-modified salts and HBDs tested to form HDESs. In green, DESs liquid at RT, in yellow the one liquid at 40-50 °C and in orange the one that did not form.

HBA \ HBD	Octanoic acid	Decanoic acid	Dodecanoic acid	Eugenol	Linoleic acid	(+/-) Menthol	Phenylpropanoic acid	Phenylacetic acid	Terpinen-4-ol	Thymol
$[N_{3\,3\,3\,(2OH)}{}^{+}][Br^{-}]$	1:4			1:2	1:4					1:2
$[N_{6\,6\,6\,(2OH)}{}^{+}][Br^{-}]$	1:4	1:2	1:2	1:2	1:2		1:3		1:2	1:2
$[N_{1\,1\,10\,(2OH)}{}^{+}][Br^{-}]$	1:3			1:3	1:2		1:2	1:2	1:2	1:2
$[N_{1\,1\,12\,(2OH)}{}^{+}][Br^{-}]$	1:3			1:3	1:3		1:2	1:2	1:4	1:2
$[N_{1\,1\,14\,(2OH)}{}^{+}][Br^{-}]$	1:3			1:3			1:3	1:3	1:4	1:2
$[N_{1\,1\,16\,(2OH)}{}^{+}][Br^{-}]$	1:4			1:4			1:3	1:3	1:4	1:2

Table S6. Retention factors of various probe molecules at 60 °C measured on deep eutectic solvents comprised of choline chloride and choline acetate hydrogen bond acceptors.

Probe	DESs				
	$[N_{1\ 1\ 10\ 2(OH)}^{+}][Br^{-}]$: 4 TEG	$[N_{1\ 1\ 16\ 2(OH)}^{+}][Br^{-}]$: 4 TEG	$[N_{3\ 3\ 3\ 2(OH)}^{+}][Br^{-}]$: 4 TEG	$[N_{4\ 4\ 4\ 4}^{+}][Br^{-}]$: 4 TEG	$[N_{6\ 6\ 6\ 2(OH)}^{+}][Br^{-}]$: 4 TEG
Methanol	0.84	0.62	1.02	1.07	0.67
Ethanol	1.01	0.80	1.21	1.33	0.88
1-Propanol	2.13	1.80	2.26	2.56	1.042
1-Butanol	4.30	3.96	4.26	5.29	5.40
1-Pentanol	8.87	8.70	7.95	10.52	11.18
Cyclopentanol	12.67	11.80	12.30	14.50	15.17
1-Hexanol	18.41	19.03	13.99	20.84	29.58
Cyclohexanol	26.14	25.92	23.29	29.00	31.94
Napthalene	48.52	55.40	49.76	59.75	61.90
Nitrobenzene	55.35	50.97	55.07	69.72	75.04
Acetophenone	44.55	40.80	44.74	51.85	54.07
Benzonitrile	28.54	25.55	30.43	37.43	36.66
Benzaldehyde	18.91	16.89	20.05	22.44	22.76
N,N-Dimethylformamide (*N,N*-DMF)	18.72	16.07	21.19	19.77	21.72
N,N-Dimethylacetamide (*N,N*-DMAC)	37.04	35.80	42.45	40.84	48.04

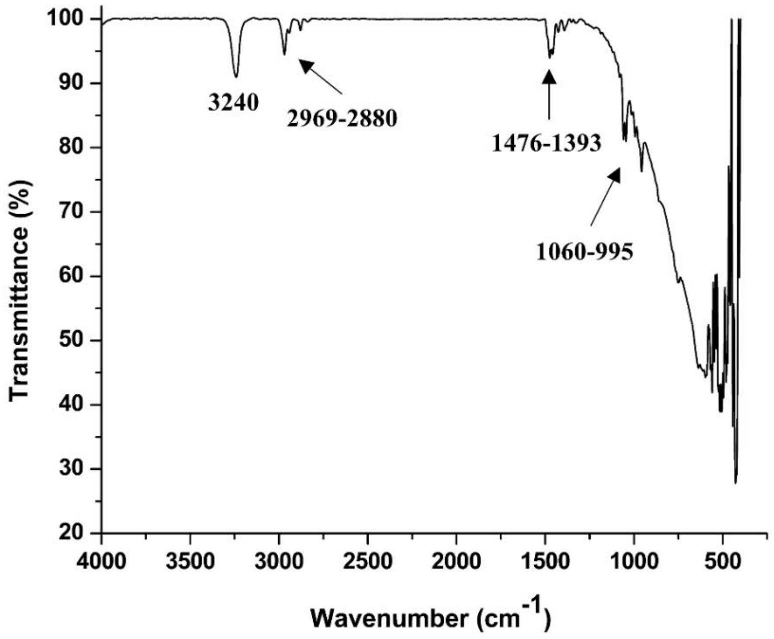

Figure S1. The ATR-FTIR spectrum for the $[N_{3\,3\,3\,2(OH)}{}^{+}][Br^{-}]$ HBA obtained under ambient conditions.

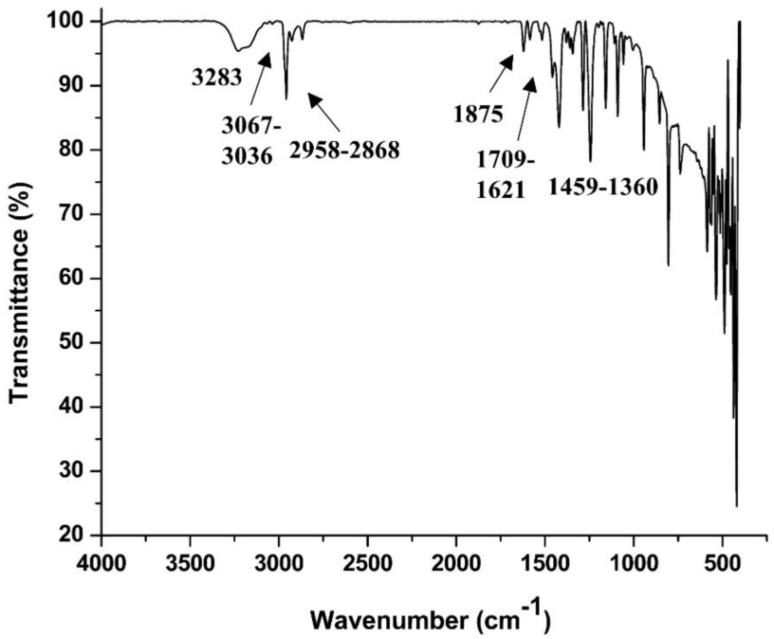

Figure S2. The ATR-FTIR spectrum for the thymol HBD obtained under ambient conditions.

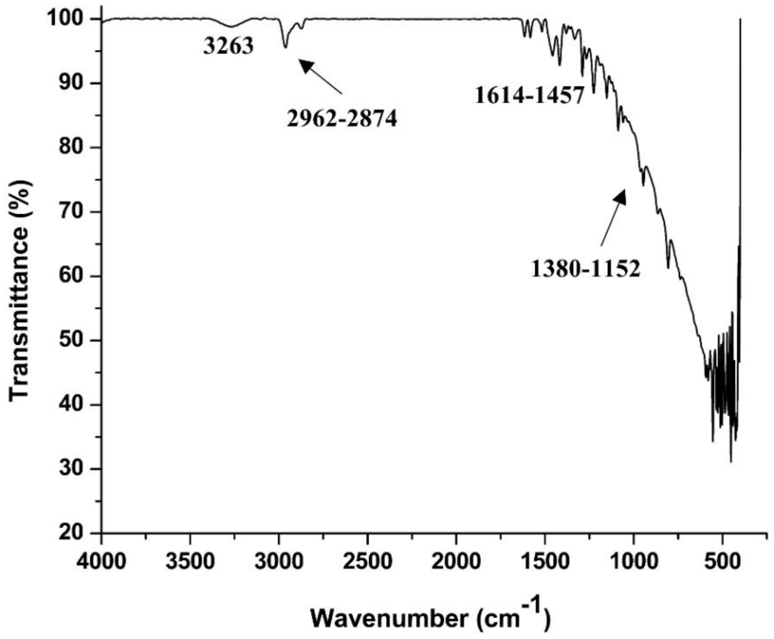

Figure S3. The ATR-FTIR spectrum for the [$N_{3\ 3\ 3\ 2(OH)}^+$][Br^-] : 2 Thymol DES obtained under ambient conditions.

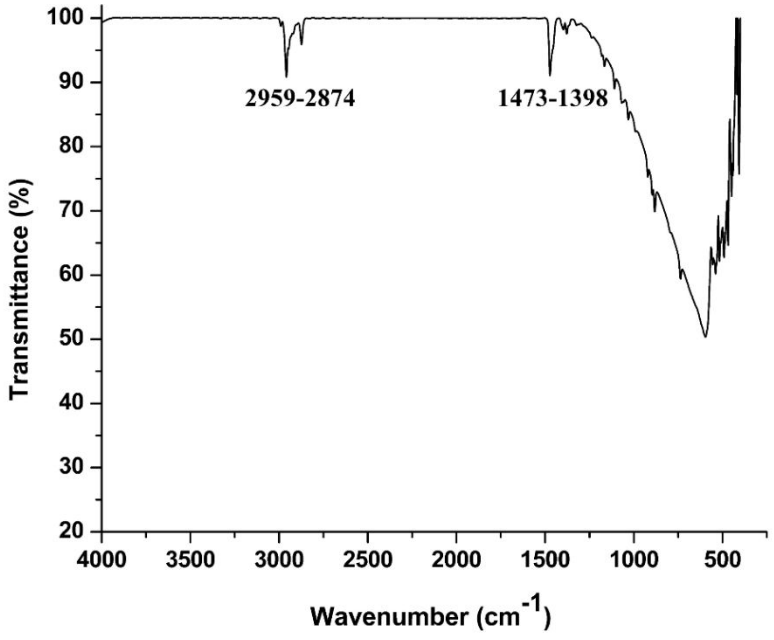

Figure S4. The ATR-FTIR spectrum for the [$N_{4\ 4\ 4\ 4}^+$][Br^-] HBA obtained under ambient conditions.

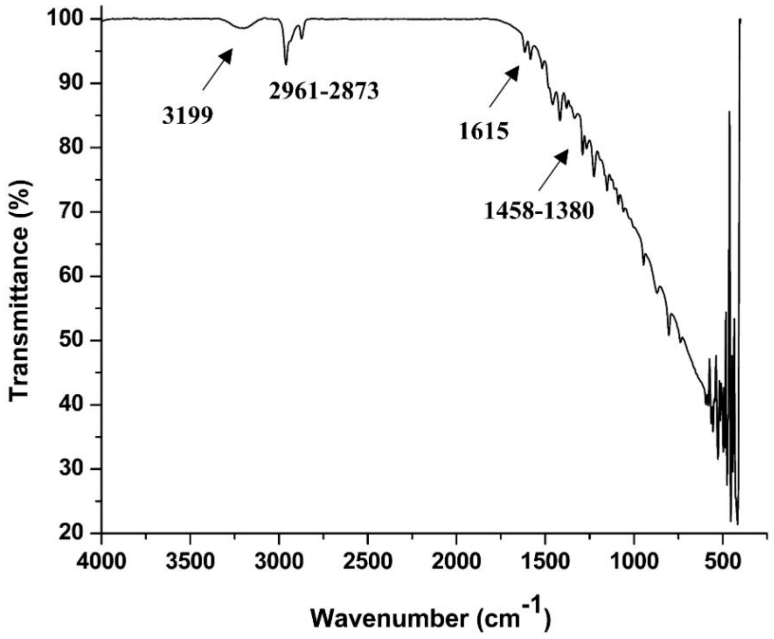

Figure S5. The ATR-FTIR spectrum for the $[N_{4\,4\,4\,4}^{+}][Br^{-}]$: 2 Thymol DES obtained under ambient conditions.

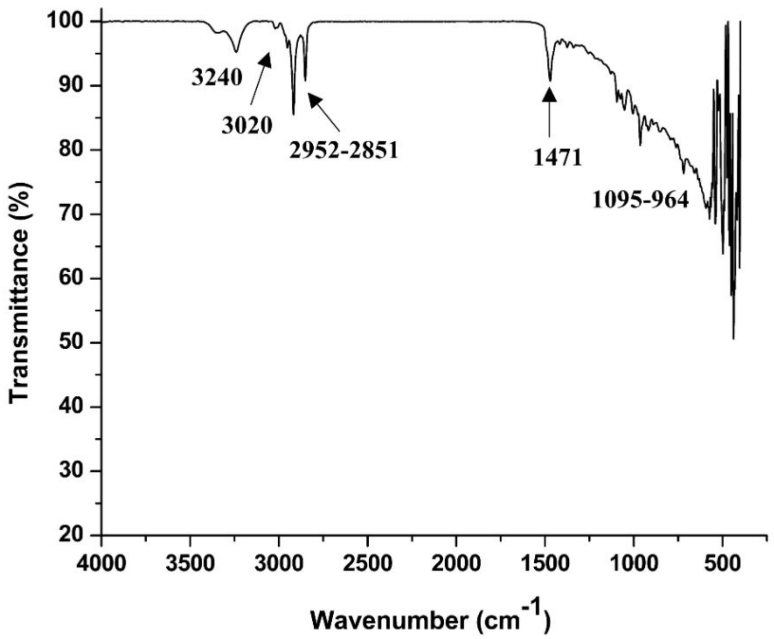

Figure S6. The ATR-FTIR spectrum for the $[N_{1\,1\,10\,2(OH)}^{+}][Br^{-}]$ HBA obtained under ambient conditions.

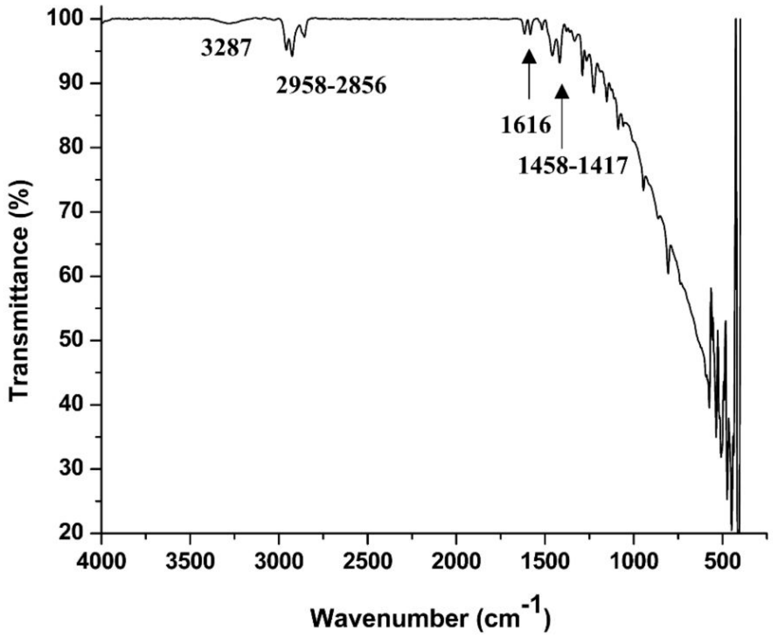

Figure S7. The ATR-FTIR spectrum for the $[N_{1\ 1\ 10\ 2(OH)}^{+}][Br^{-}]$: 2 Thymol DES obtained under ambient conditions.

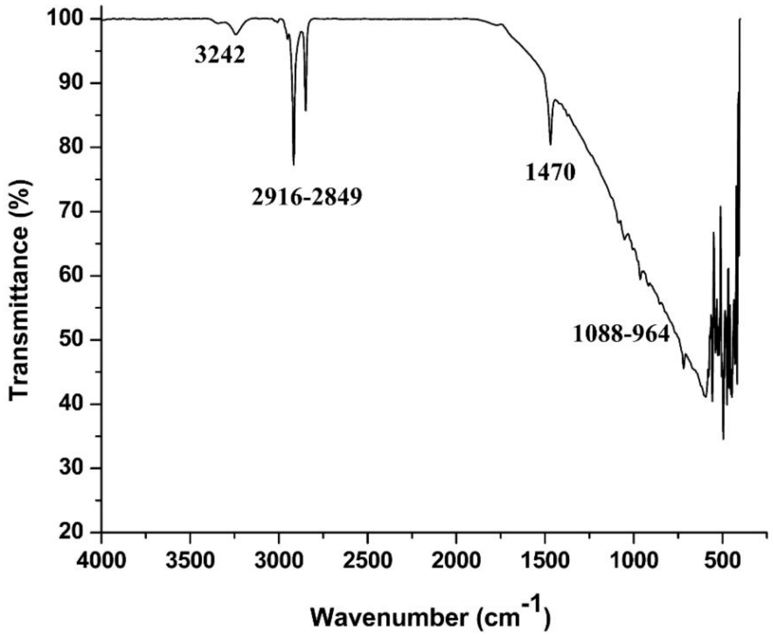

Figure S8. The ATR-FTIR spectrum for the $[N_{1\ 1\ 16\ 2(OH)}^{+}][Br^{-}]$ HBA obtained under ambient conditions.

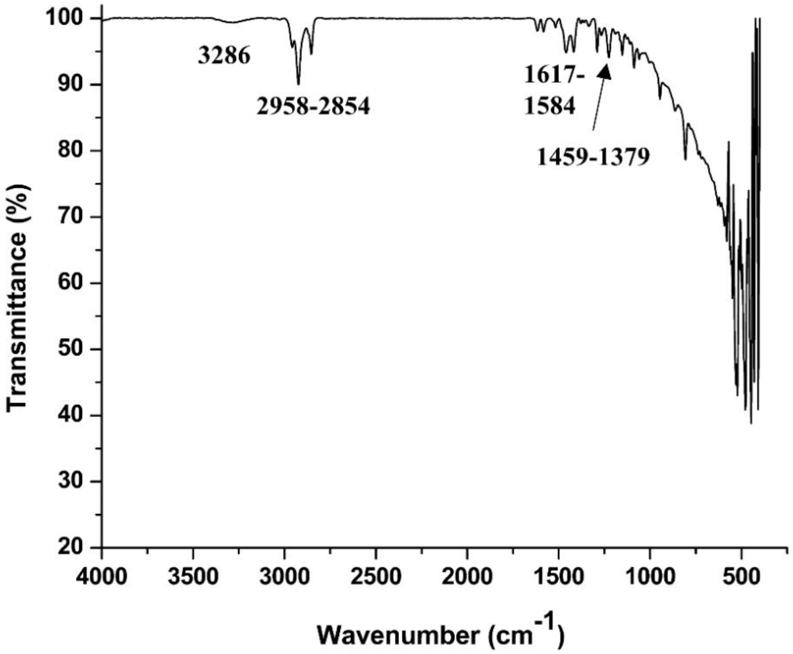

Figure S9. The ATR-FTIR spectrum for the $[N_{1\ 1\ 16\ 2(OH)}{}^{+}][Br^{-}]$: 2 Thymol DES obtained under ambient conditions.

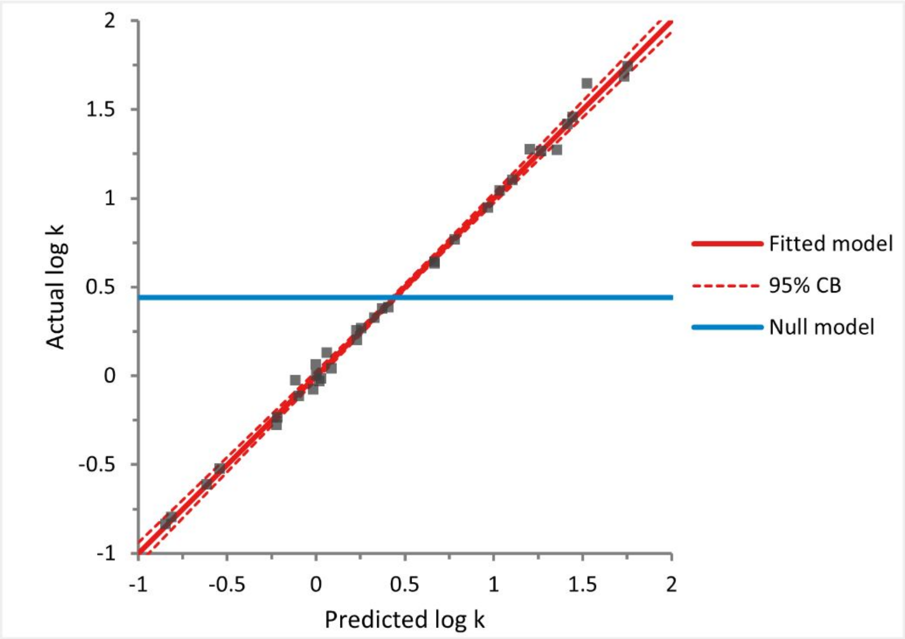

Figure S10. Multiple linear regression plot ($R^2 = 0.99$) for the $[N_{1\ 1\ 10\ 2(OH)}{}^{+}][Br^{-}]$: 4 TEG DES at 60 °C. Solute descriptors and retention factors of 35 analytes are evaluated by multiple linear regression analysis to generate the system constants for the Abraham solvation parameter model (equation 5).

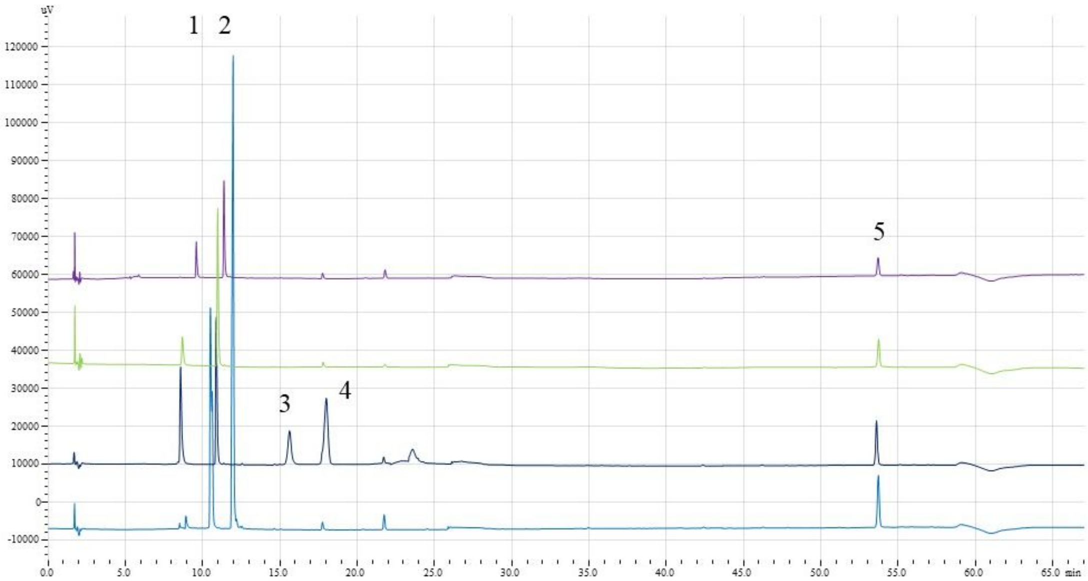

Figure S11. Chromatographic profiles at 340 nm of the DSLME of luteolin-7-*O*-glucuronide, apigenin-7-*O*-glucuronide and CBDA standard compounds, using $[N_{1\,1\,10\,(2OH)}{}^{+}][Br^{-}]$: 2 Thymol (light blue), $[N_{1\,1\,16\,(2OH)}{}^{+}][Br^{-}]$: 2 Thymol (blue), $[N_{3\,3\,3\,(2OH)}{}^{+}][Br^{-}]$: 2 Thymol (green) and $[N_{4\,4\,4\,4}{}^{+}][Br^{-}]$: 2 Thymol (purple) as solvents. Peaks identification: 1. luteolin-7-*O*-glucuronide, 2. apigenin-7-*O*-glucuronide, 3. luteolin, 4. apigenin, 5. CBDA. The shift of the peaks in the first part of the chromatogram is due to the interference given by the salts used as HBAs.

NMR Spectra

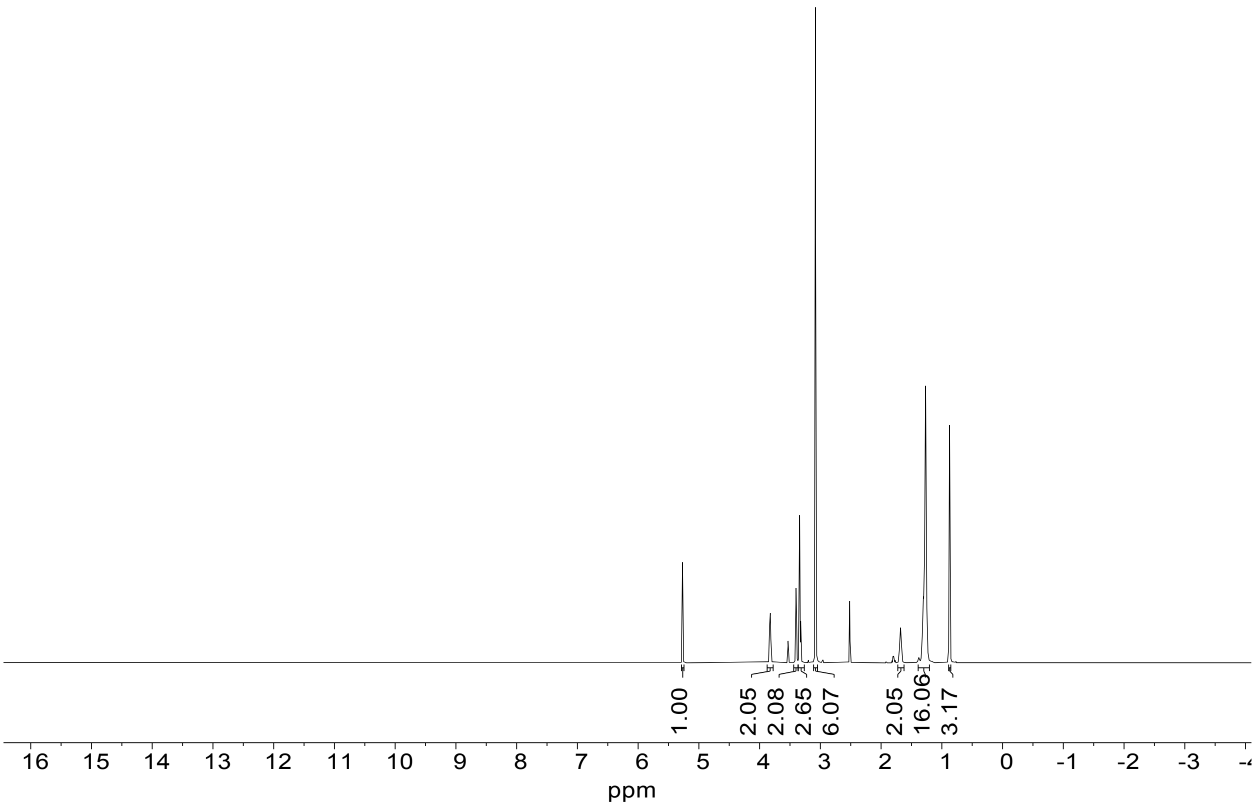

Figure S12: ^{1}H NMR of [N $_{1\,1\,10\,(2OH)}$$^{+}$][Br^{-}]

^{1}H NMR (600 MHz, DMSO) δ 5.27 (t, *J* = 5.0 Hz, 1H), 3.83 (tdd, *J* = 7.0, 4.9, 2.5 Hz, 2H), 3.44 – 3.36 (m, 2H), 3.37 – 3.26 (m, 3H), 3.08 (s, 6H), 1.73 – 1.62 (m, 2H), 1.29 (d, *J* = 21.6 Hz, 16H), 0.87 (td, *J* = 7.0, 2.5 Hz, 3H).

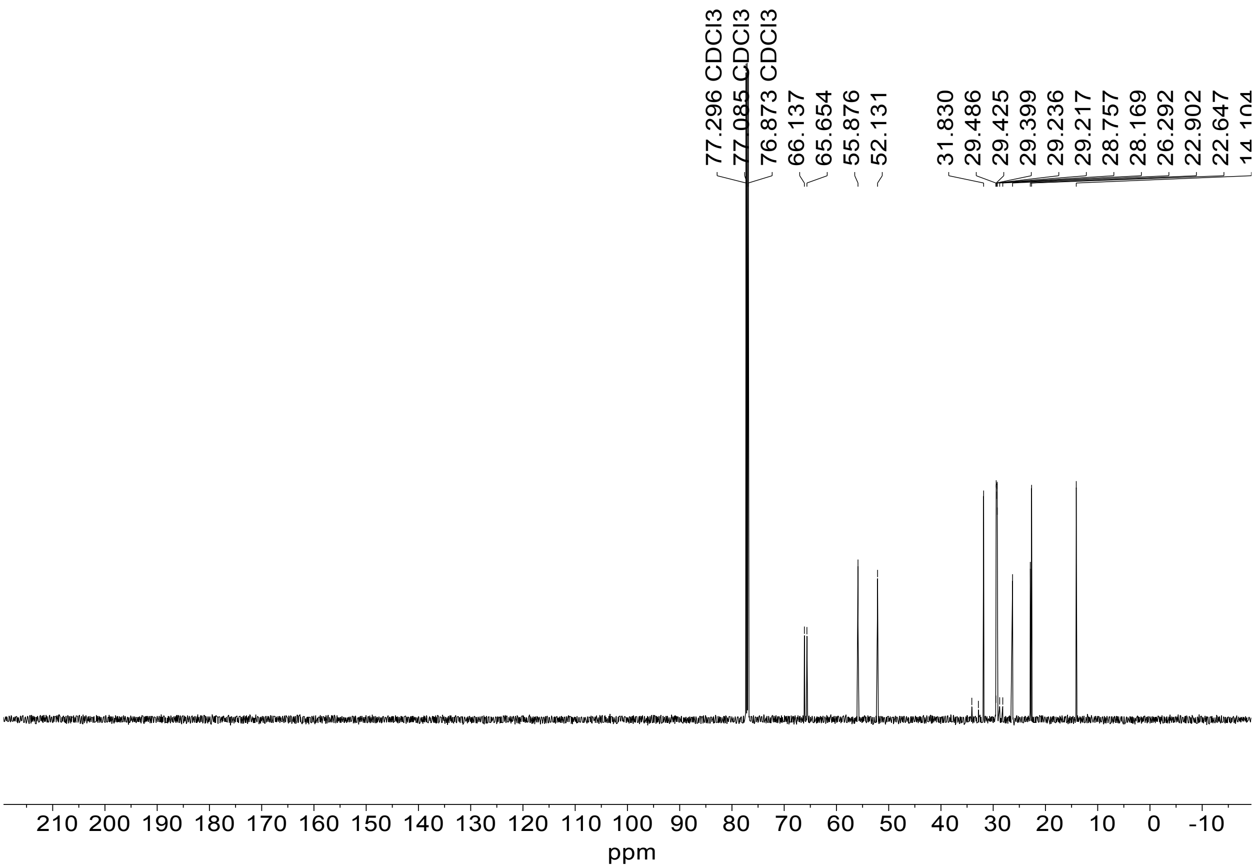

Figure S13: ^{13}C NMR of [N $_{1\,1\,10\,(2OH)}$$^{+}$][Br^{-}]

^{13}C NMR (151 MHz, $CDCl_3$) δ 66.14, 65.65, 55.88, 52.13, 31.83, 29.43, 29.40, 29.24, 29.22, 28.76, 26.29, 22.90, 22.65, 14.10.

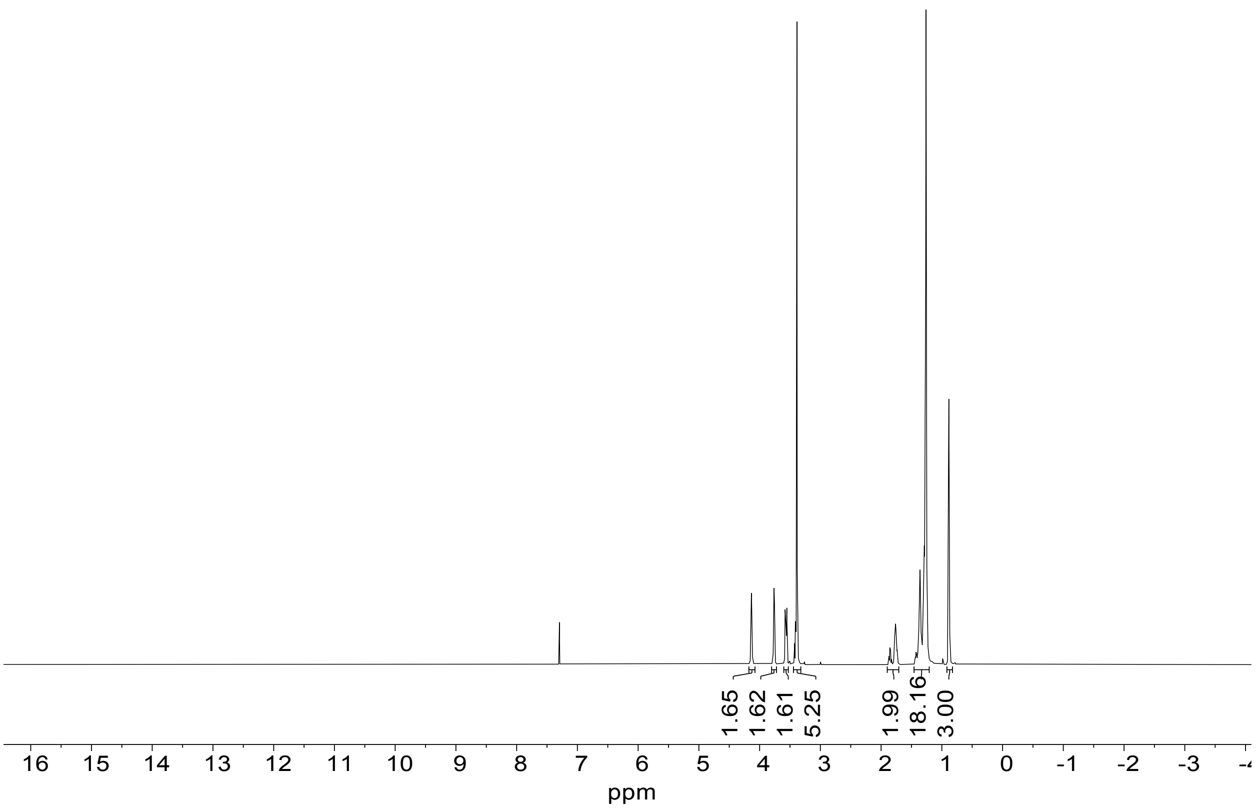

Figure S14: ^{1}H NMR of [N $_{1\,1\,12\,(2OH)}$$^{+}$][Br^{-}]

^{1}H NMR (600 MHz, $CDCl_3$) δ 4.18 – 4.08 (m, 2H), 3.80 – 3.72 (m, 2H), 3.60 – 3.53 (m, 2H), 3.39 (s, 5H), 1.90 – 1.71 (m, 2H), 1.46 – 1.21 (m, 18H), 0.88 (t, J = 7.0 Hz, 3H).

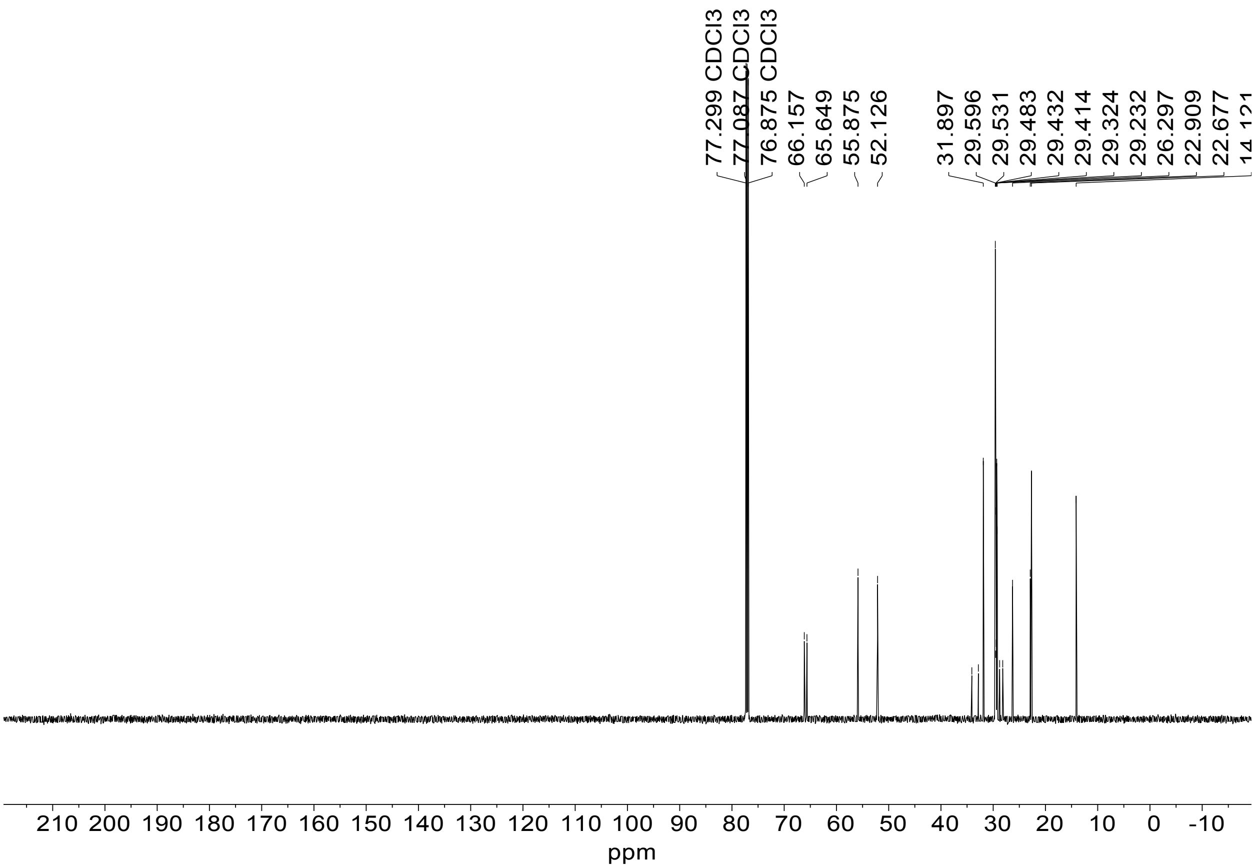

Figure S15: ^{13}C NMR of [N $_{1\,1\,12\,(2OH)}$$^{+}$][Br^{-}]

^{13}C NMR (151 MHz, $CDCl_3$) δ 66.16, 65.65, 55.88, 52.13, 34.09, 32.84, 31.90, 29.60, 29.53, 29.48, 29.43, 29.41, 29.32, 29.23, 28.76, 28.17, 26.30, 22.91, 22.68, 14.12.

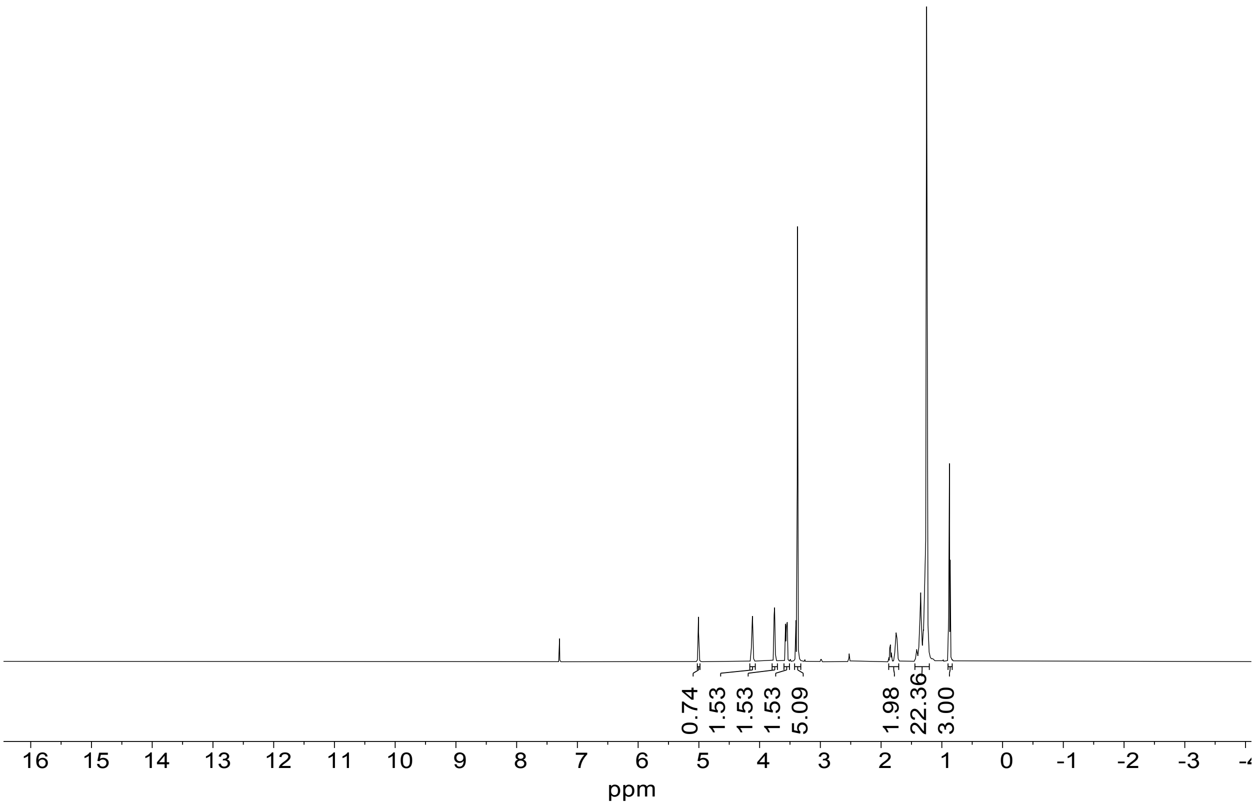

Figure S16: ^{1}H NMR of [N $_{1\,1\,14\,(2OH)}$$^+$][Br$^-$]

^{1}H NMR (600 MHz, $CDCl_3$) δ 5.01 (t, *J* = 5.5 Hz, 1H), 4.12 (q, *J* = 5.0 Hz, 2H), 3.79 – 3.71 (m, 2H), 3.60 – 3.51 (m, 2H), 3.38 (s, 5H), 1.87 – 1.71 (m, 2H), 1.44 – 1.21 (m, 22H), 0.87 (t, *J* = 7.0 Hz, 3H).

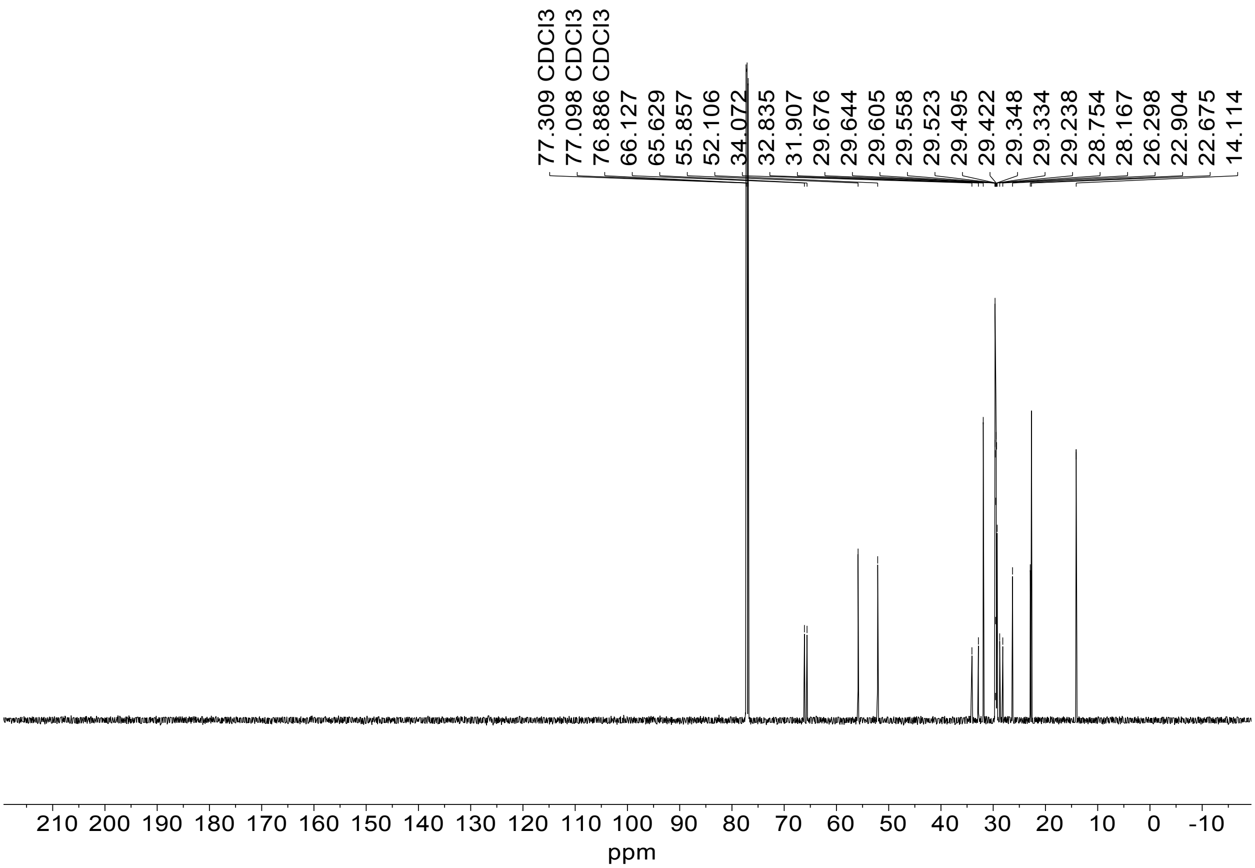

Figure S17: ^{13}C NMR of [N $_{1\ 1\ 14\ (2OH)}$$^{+}$][Br^{-}]

^{13}C NMR (151 MHz, $CDCl_3$) δ 66.13, 65.63, 55.86, 52.11, 34.07, 32.84, 31.91, 29.68, 29.64, 29.60, 29.52, 29.50, 29.42, 29.35, 29.33, 29.24, 28.75, 28.17, 26.30, 22.90, 22.67, 14.11.

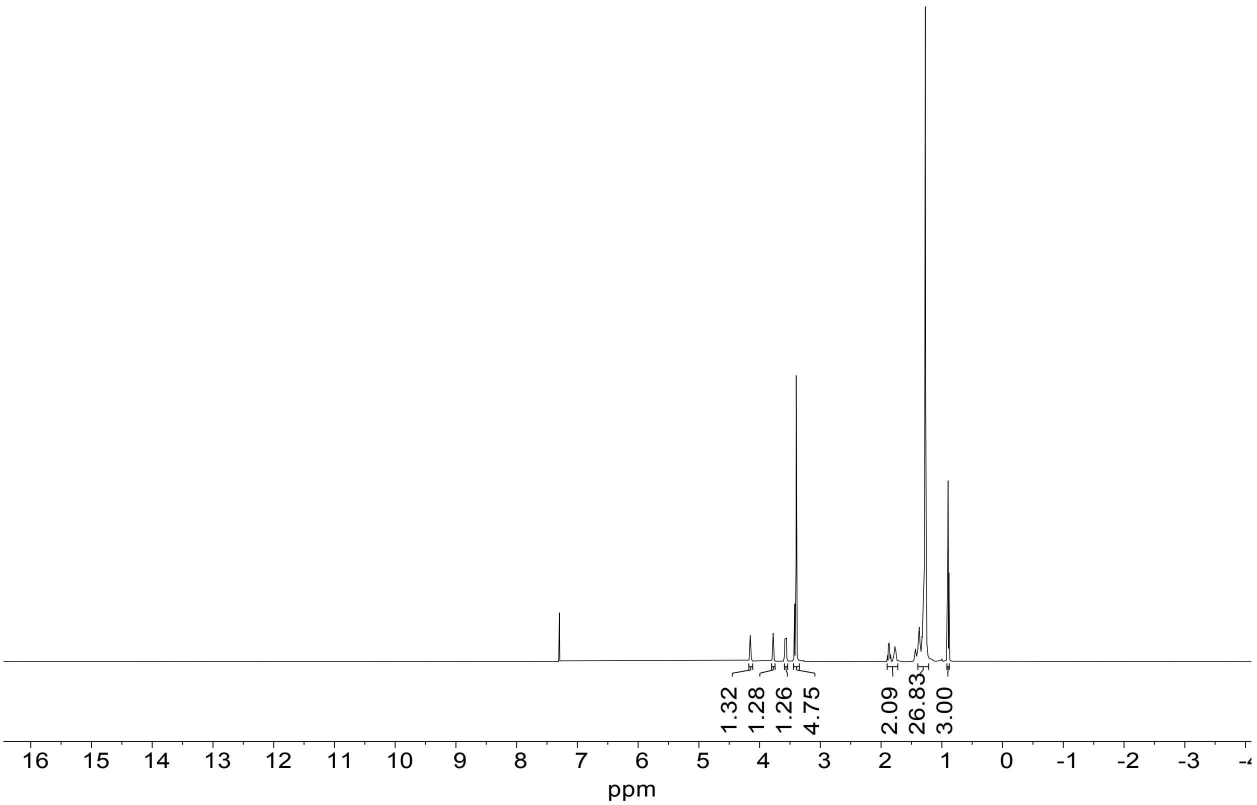

Figure S18: ^{1}H NMR of [N $_{1\ 1\ 16\ (2OH)}$$^{+}$][Br^{-}]

^{1}H NMR (600 MHz, $CDCl_3$) δ 4.18 – 4.11 (m, 1H), 3.80 – 3.75 (m, 1H), 3.59 – 3.54 (m, 1H), 3.41 (d, *J* = 15.0 Hz, 5H), 1.90 – 1.72 (m, 2H), 1.40 – 1.22 (m, 27H), 0.90 (t, *J* = 7.0 Hz, 3H).

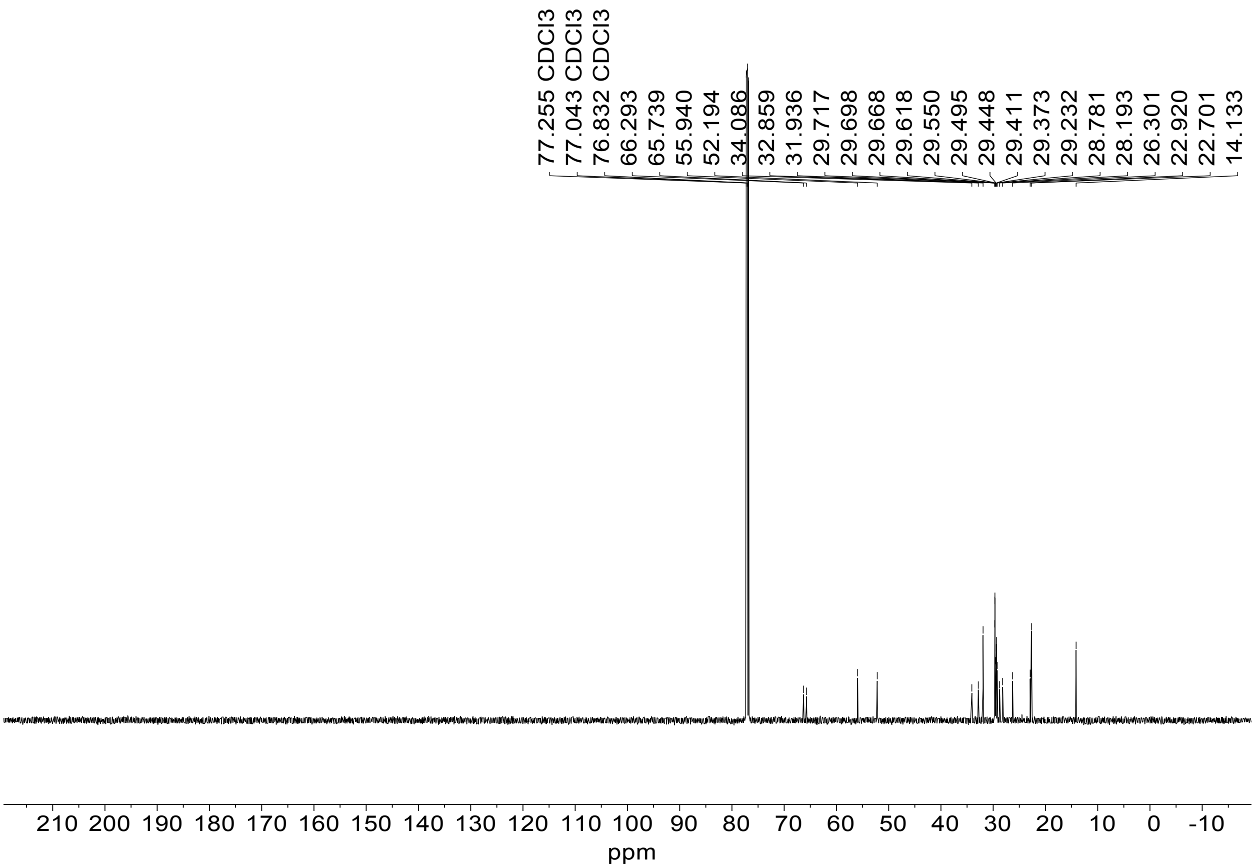

Figure S19: ^{13}C NMR of [N $_{1\,1\,16\,(2OH)}$$^{+}$][Br^{-}]

^{13}C NMR (151 MHz, $CDCl_3$) δ 66.29, 65.74, 55.94, 52.19, 34.09, 32.86, 31.94, 29.72, 29.70, 29.67, 29.62, 29.55, 29.49, 29.45, 29.41, 29.37, 29.23, 28.78, 28.19, 26.30, 22.92, 22.70, 14.13.

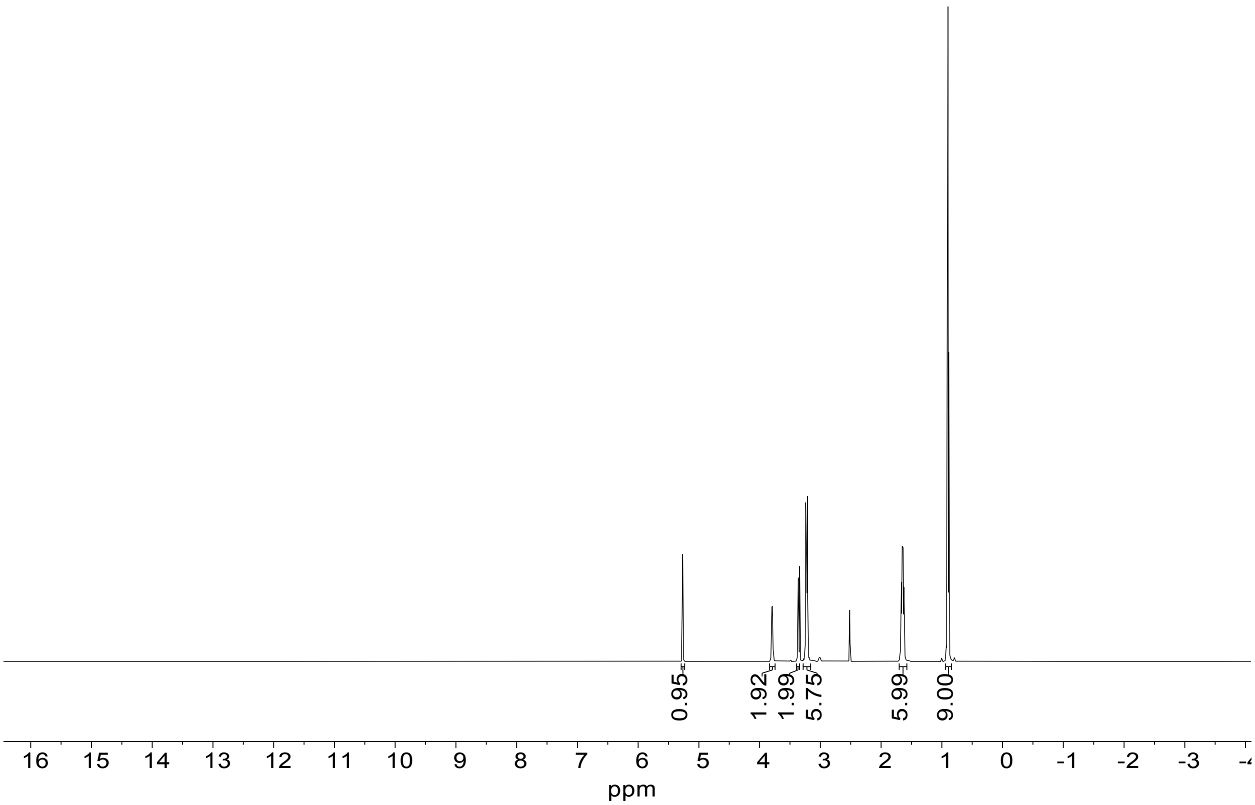

Figure S20: ^{1}H NMR of [N $_{3\,3\,3\,(2OH)}$$^{+}$][Br^{-}]

^{1}H NMR (600 MHz, DMSO) δ 5.27 (t, *J* = 5.2 Hz, 1H), 3.79 (q, *J* = 4.9 Hz, 2H), 3.36 (dd, *J* = 6.1, 4.2 Hz, 2H), 3.28 – 3.16 (m, 6H), 1.70 – 1.58 (m, 6H), 0.90 (t, *J* = 7.3 Hz, 9H).

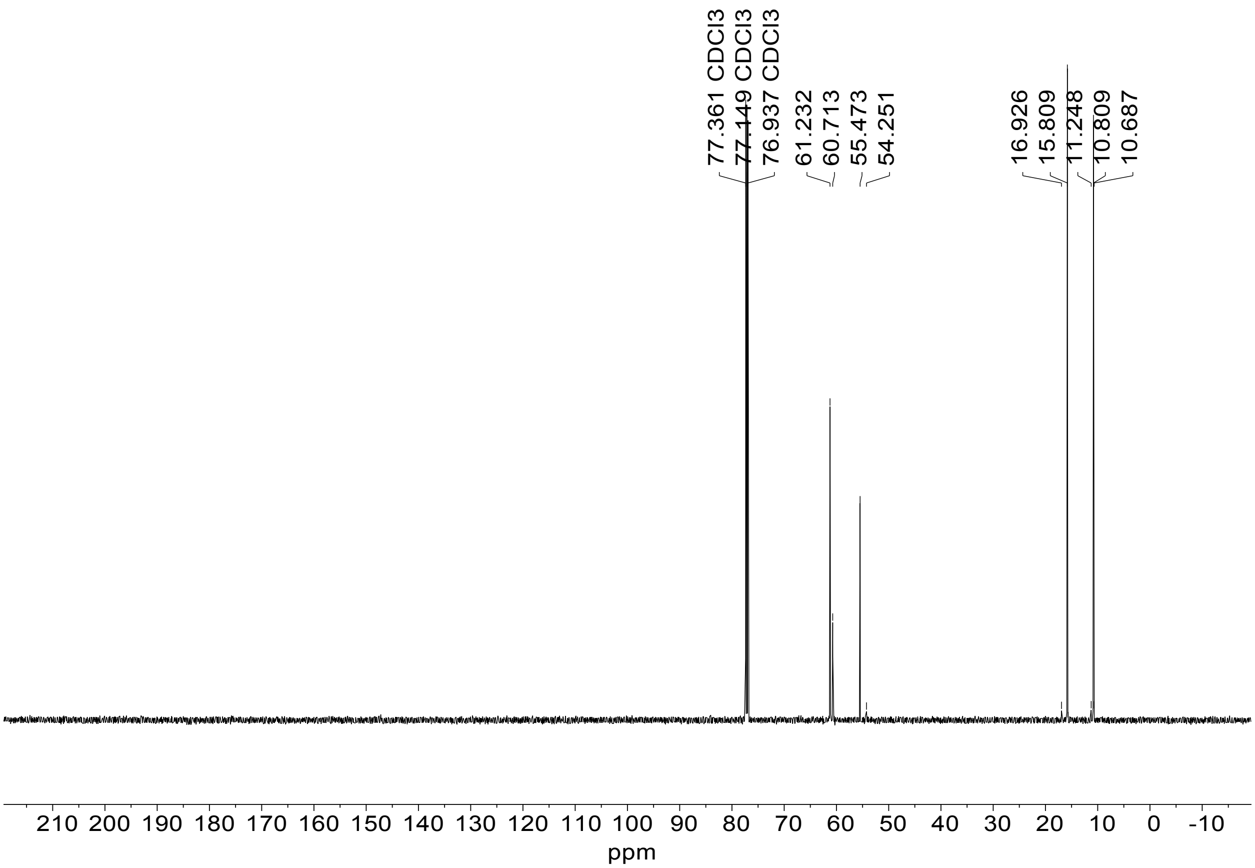

Figure S21: ^{13}C NMR of $[N_{3\,3\,3\,(2OH)}{}^{+}][Br^{-}]$

^{13}C NMR (151 MHz, $CDCl_3$) δ 61.23, 60.71, 55.47, 15.81, 10.81.

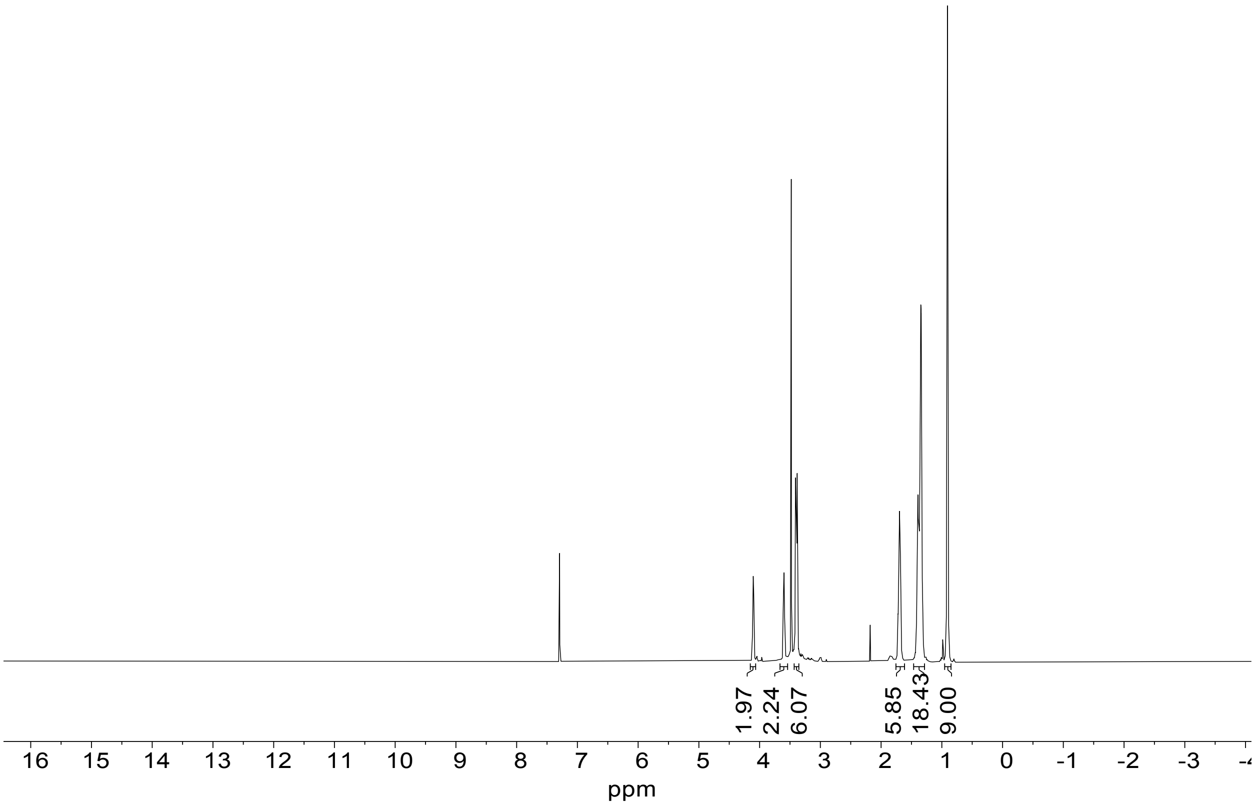

Figure S22: ^{1}H NMR of [N $_{6\,6\,6\,(2OH)}$$^+$][Br$^-$]

^{1}H NMR (600 MHz, $CDCl_3$) δ 4.11 (t, *J* = 4.6 Hz, 2H), 3.66 – 3.54 (m, 2H), 3.43 – 3.35 (m, 6H), 1.76 – 1.61 (m, 6H), 1.46 – 1.28 (m, 18H), 0.95 – 0.85 (m, 9H).

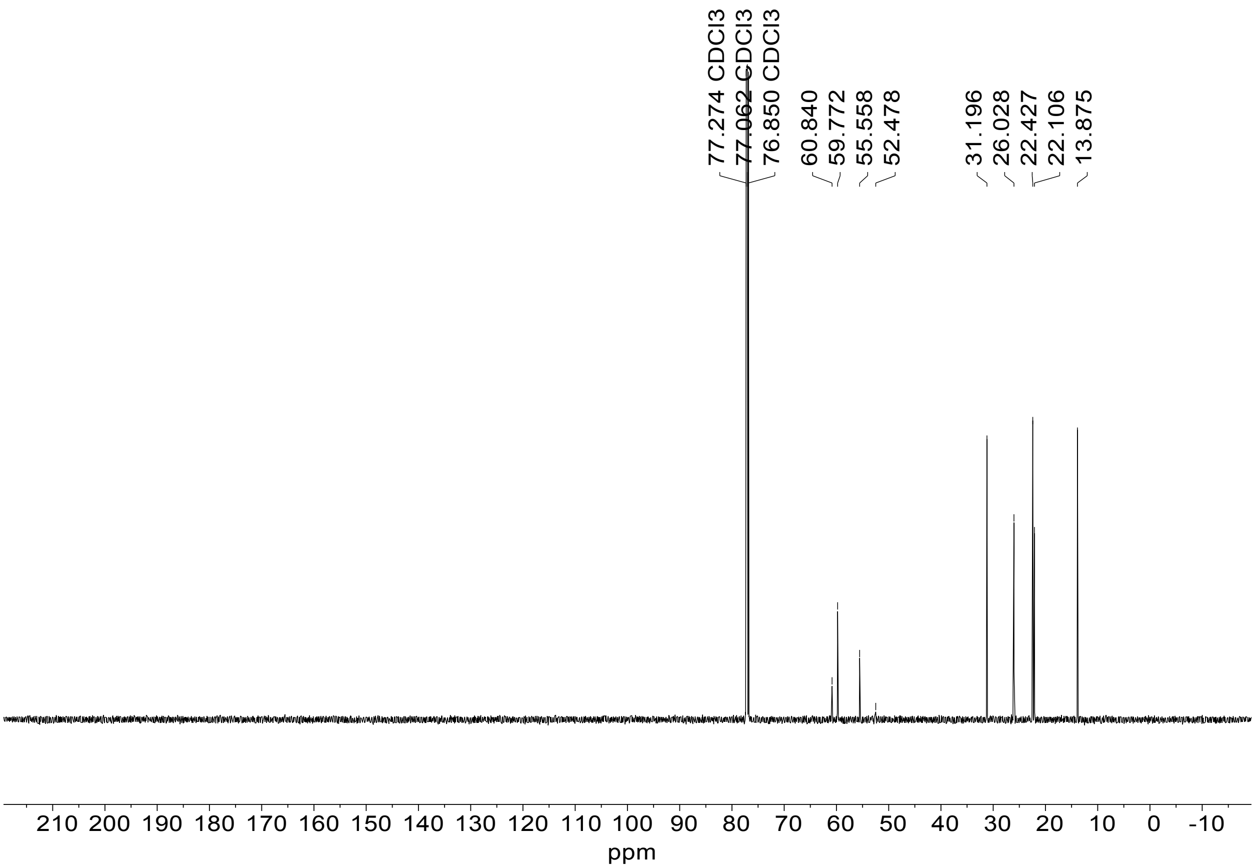

Figure S23: ^{13}C NMR of [N $_{6\,6\,6\,(2OH)}$$^{+}$][Br^{-}]

^{13}C NMR (151 MHz, $CDCl_3$) δ 60.84, 59.77, 55.56, 31.20, 26.03, 22.43, 22.11, 13.88.

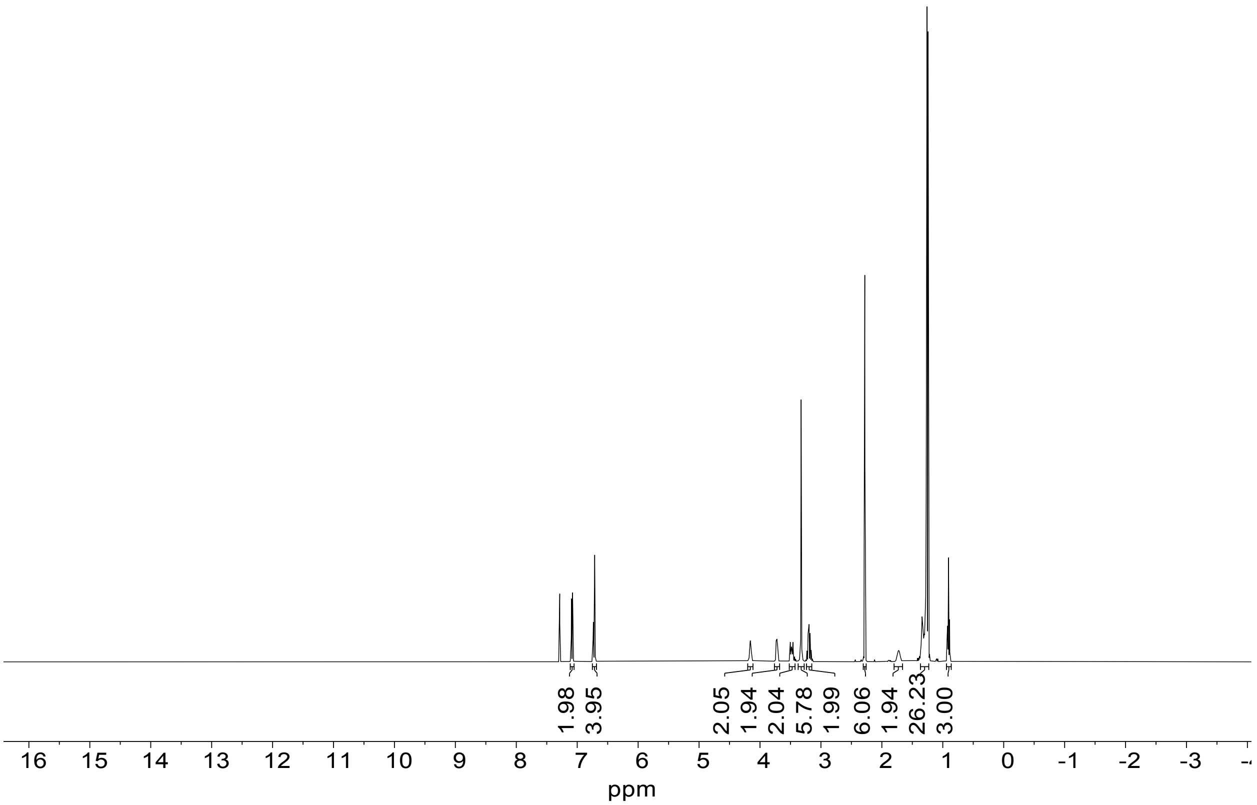

Figure S24: ^{1}H NMR of [N $_{1\,1\,10\,(2OH)}$$^+$][Br$^-$] : 2 Thymol HDES

^{1}H NMR (400 MHz, $CDCl_3$) δ 7.09 (d, *J* = 7.6 Hz, 2H), 6.75 – 6.68 (m, 4H), 4.16 (t, *J* = 4.7 Hz, 2H), 3.76 – 3.68 (m, 2H), 3.52 – 3.42 (m, 2H), 3.32 (s, 6H), 3.19 (p, *J* = 6.9 Hz, 2H), 2.28 (s, 6H), 1.80 – 1.66 (m, 2H), 1.37 – 1.23 (m, 26H), 0.91 (t, *J* = 6.9 Hz, 3H).

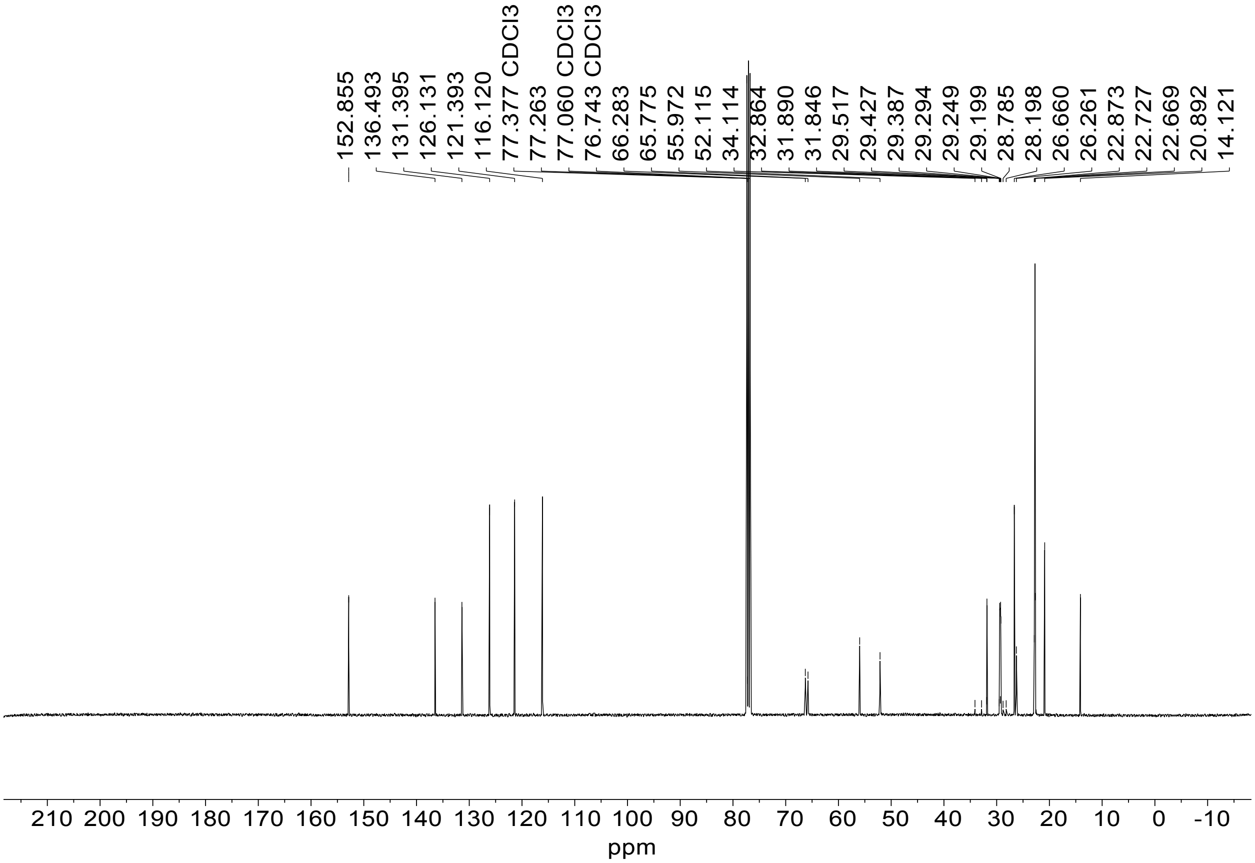

Figure S25: ^{13}C NMR of $[N_{1\ 1\ 10\ (2OH)}{}^{+}][Br^{-}]$: 2 Thymol HDES

^{13}C NMR (101 MHz, $CDCl_3$) δ 152.86, 136.49, 131.39, 126.13, 121.39, 116.12, 77.26, 66.28, 65.78, 55.97, 52.11, 31.85, 29.43, 29.39, 29.25, 29.20, 26.66, 26.26, 22.87, 22.73, 22.67, 20.89, 14.12.

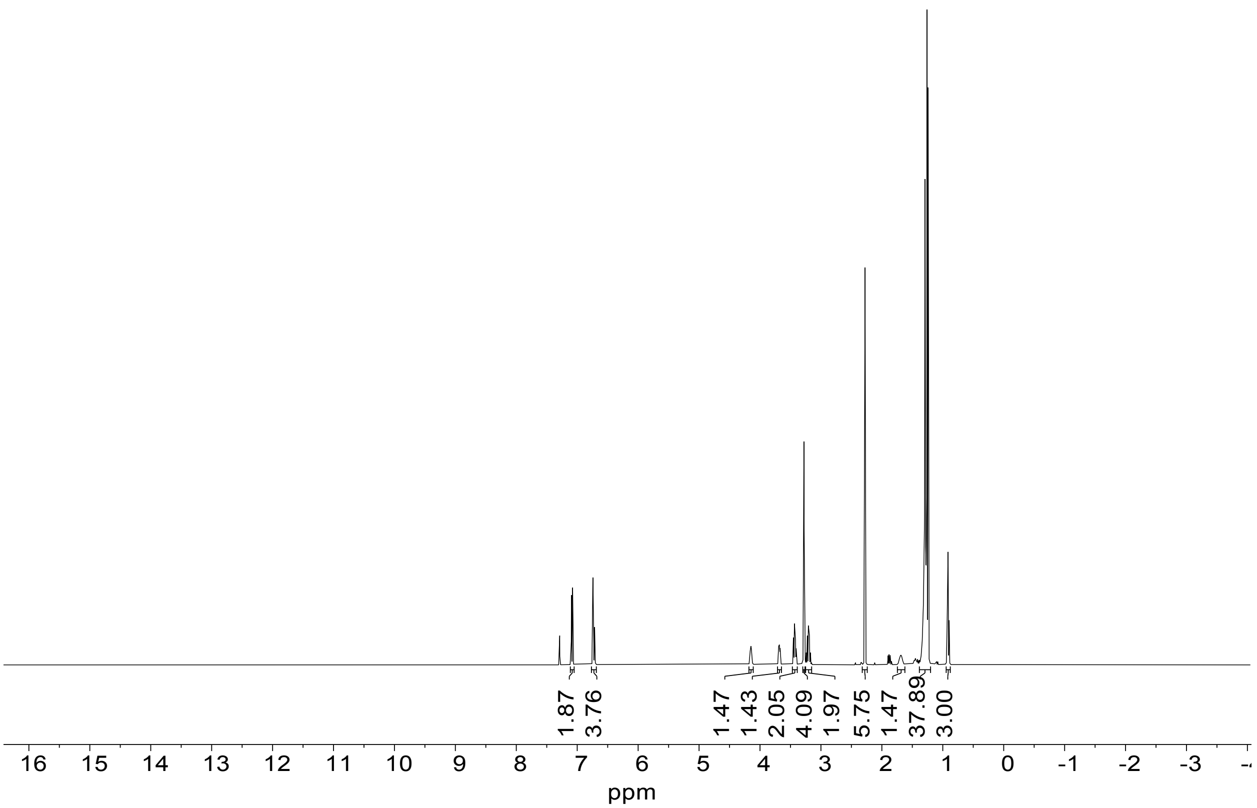

Figure S26: ^{1}H NMR of [N $_{1\,1\,16\,(2OH)}$$^+$][Br$^-$] : 2 Thymol HDES

^{1}H NMR (400 MHz, $CDCl_3$) δ 7.09 (d, *J* = 7.7 Hz, 2H), 6.77 – 6.69 (m, 4H), 4.18 – 4.11 (m, 1H), 3.71 – 3.65 (m, 1H), 3.47 – 3.39 (m, 2H), 3.28 (s, 4H), 3.20 (h, *J* = 6.9 Hz, 2H), 2.28 (s, 6H), 1.69 (dq, *J* = 10.0, 5.5 Hz, 1H), 1.38 – 1.20 (m, 38H), 0.94 – 0.88 (m, 3H).

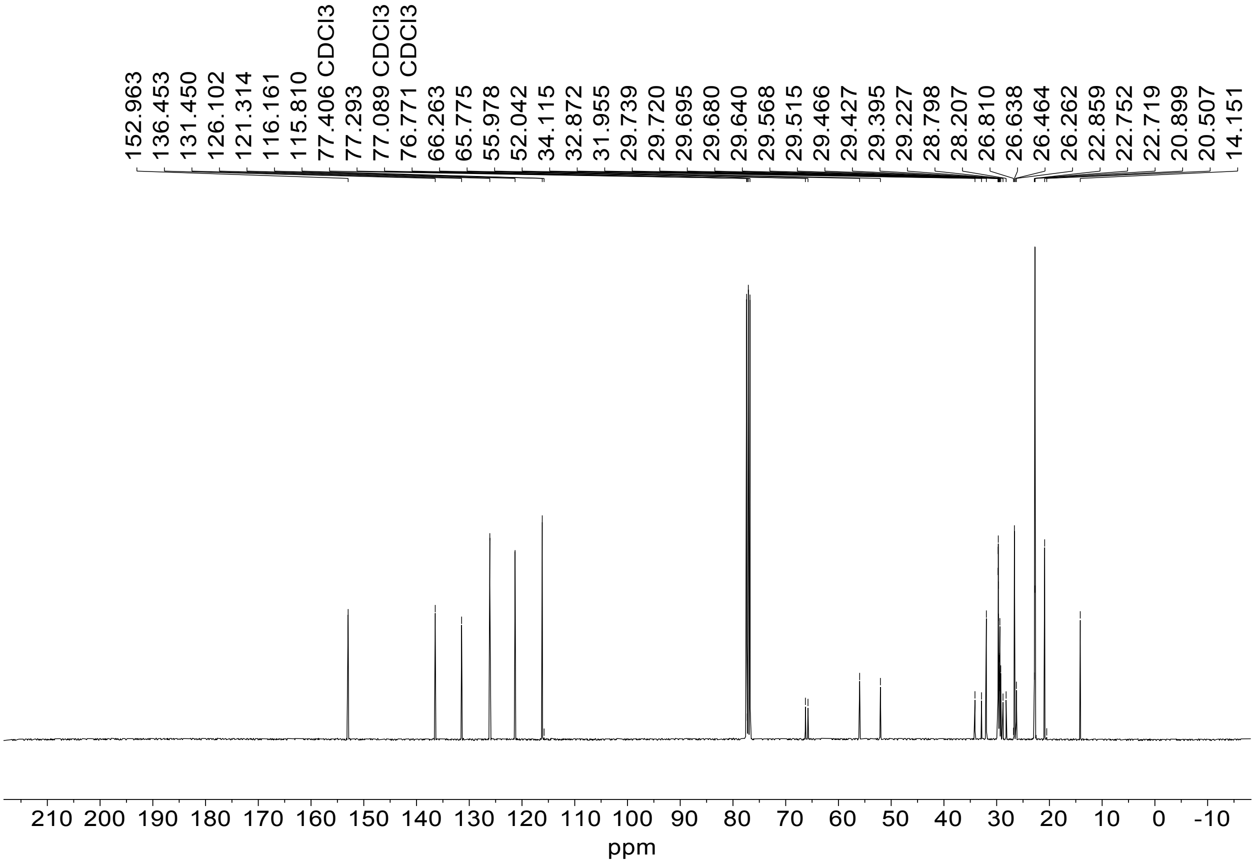

Figure S27: ^{13}C NMR of $[N_{1\,1\,16\,(2OH)}{}^{+}][Br^{-}]$: 2 Thymol HDES

^{13}C NMR (101 MHz, $CDCl_3$) δ 152.96, 136.45, 131.45, 126.10, 121.31, 116.16, 77.29, 66.26, 65.78, 55.98, 52.04, 34.12, 32.87, 31.95, 29.74, 29.72, 29.69, 29.68, 29.64, 29.57, 29.52, 29.47, 29.43, 29.39, 29.23, 28.80, 28.21, 26.64, 26.26, 22.86, 22.75, 22.72, 20.90, 14.15.

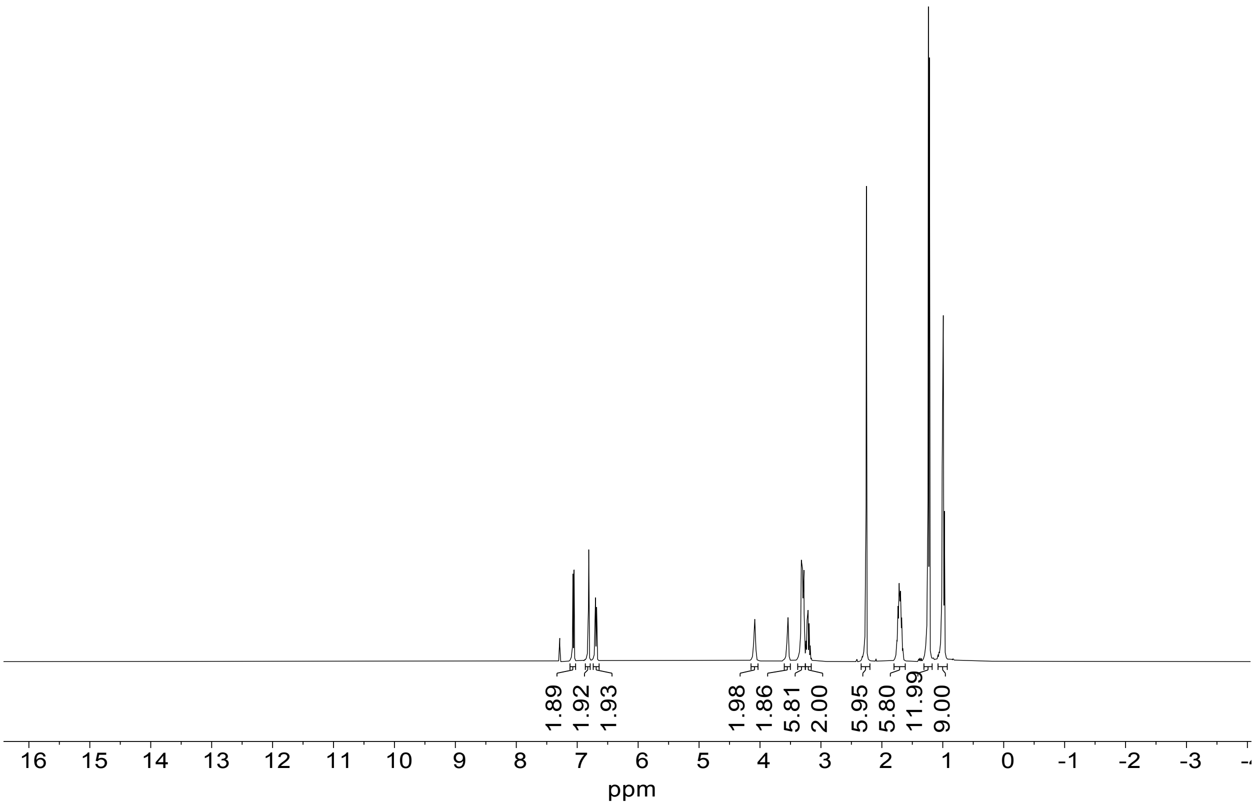

Figure S28: ^{1}H NMR of [N $_{3\,3\,3\,(2OH)}$$^+$][Br$^-$] : 2 Thymol HDES

^{1}H NMR (400 MHz, $CDCl_3$) δ 7.06 (d, *J* = 7.7 Hz, 2H), 6.81 (d, *J* = 1.7 Hz, 2H), 6.69 (dd, *J* = 7.8, 1.8 Hz, 2H), 4.15 – 4.03 (m, 2H), 3.60 – 3.50 (m, 2H), 3.38 – 3.26 (m, 6H), 3.20 (h, *J* = 6.9 Hz, 2H), 2.25 (s, 6H), 1.80 – 1.62 (m, 6H), 1.23 (d, *J* = 7.0 Hz, 12H), 0.99 (t, *J* = 7.2 Hz, 9H).

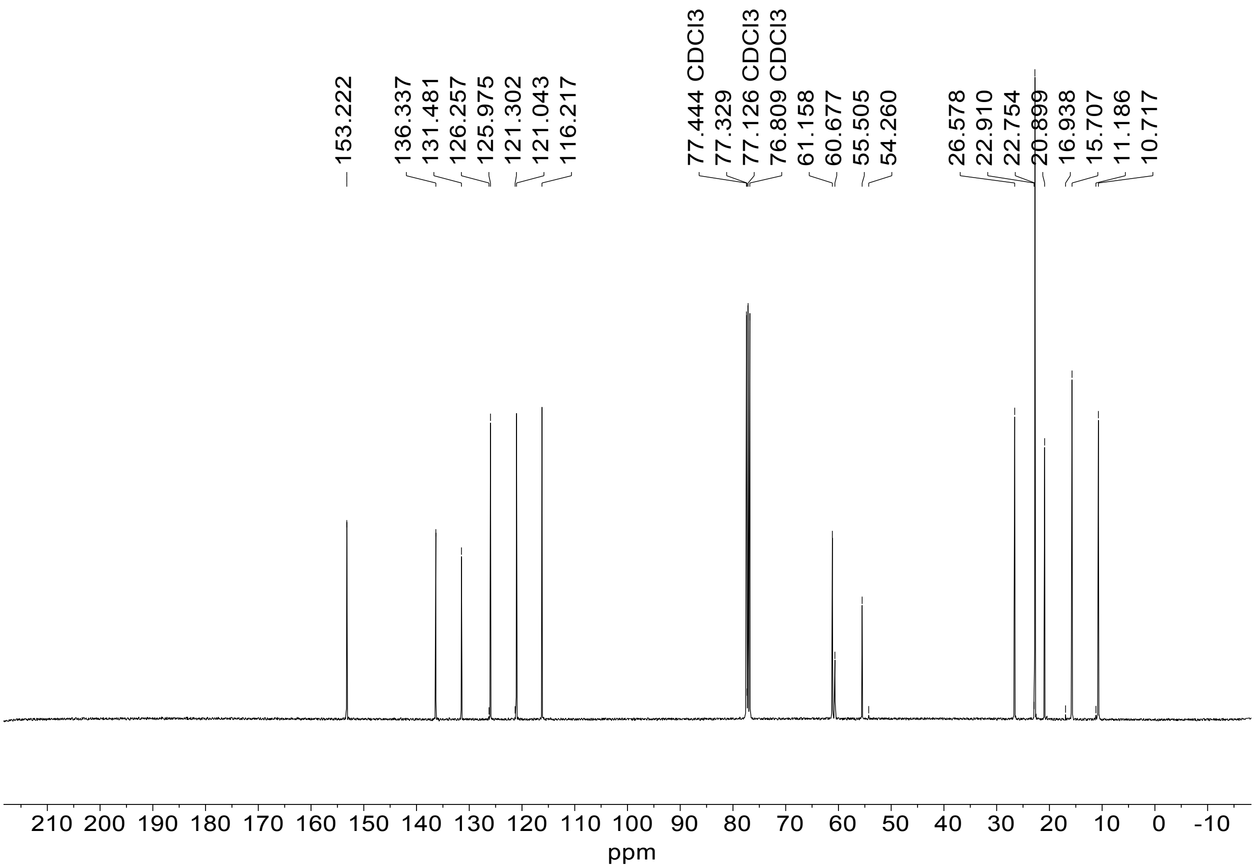

Figure S29: ^{13}C NMR of $[N_{3\,3\,3\,(2OH)}{}^{+}][Br^{-}]$: 2 Thymol HDES

^{13}C NMR (101 MHz, $CDCl_3$) δ 153.22, 136.34, 131.48, 125.97, 121.04, 116.22, 61.16, 60.68, 55.50, 26.58, 22.75, 20.90, 15.71, 10.72.

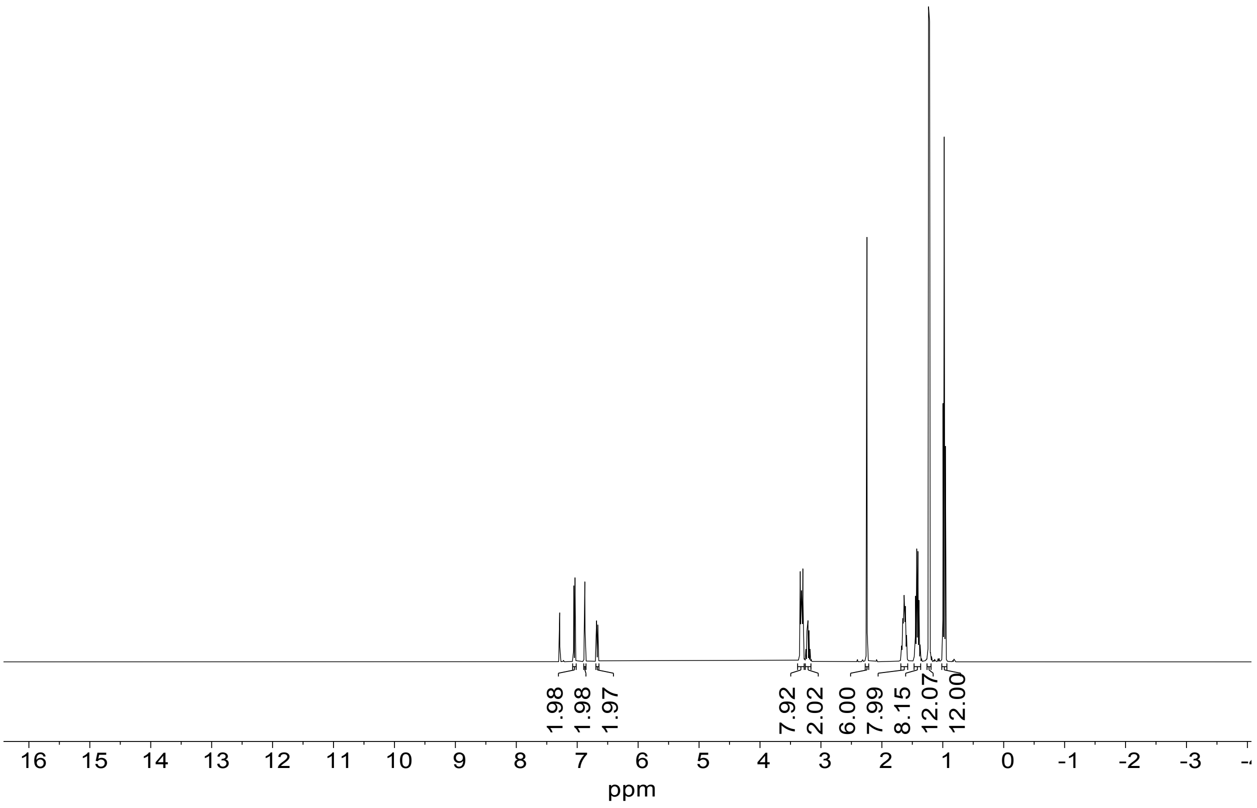

Figure S30: ^{1}H NMR of [N $_{4444}$$^{+}$][Br^{-}] : 2 Thymol HDES

^{1}H NMR (400 MHz, $CDCl_3$) δ 7.05 (d, *J* = 7.7 Hz, 2H), 6.88 (dd, *J* = 1.7, 0.8 Hz, 2H), 6.69 – 6.64 (m, 2H), 3.38 – 3.28 (m, 8H), 3.21 (p, *J* = 7.0 Hz, 2H), 2.25 (s, 6H), 1.63 (dq, *J* = 11.9, 7.6 Hz, 8H), 1.42 (h, *J* = 7.4 Hz, 8H), 1.22 (d, *J* = 6.9 Hz, 12H), 0.98 (t, *J* = 7.3 Hz, 12H).

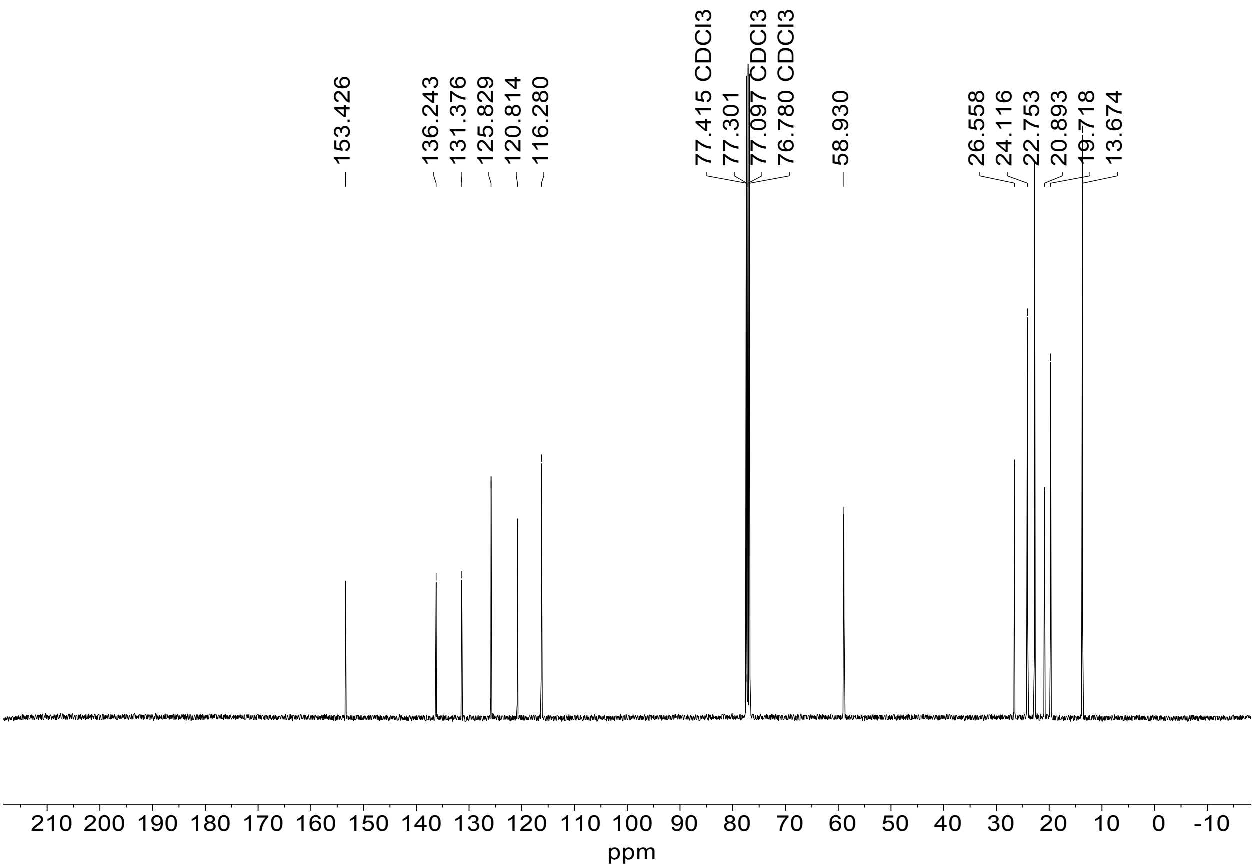

Figure S32: ^{13}C NMR of [N $_{4444}$$^{+}$][Br^{-}] : 2 Thymol HDES

^{13}C NMR (101 MHz, $CDCl_3$) δ 153.43, 136.24, 131.38, 125.83, 120.81, 116.28, 77.30, 58.93, 26.56, 24.12, 22.75, 20.89, 19.72, 13.67.

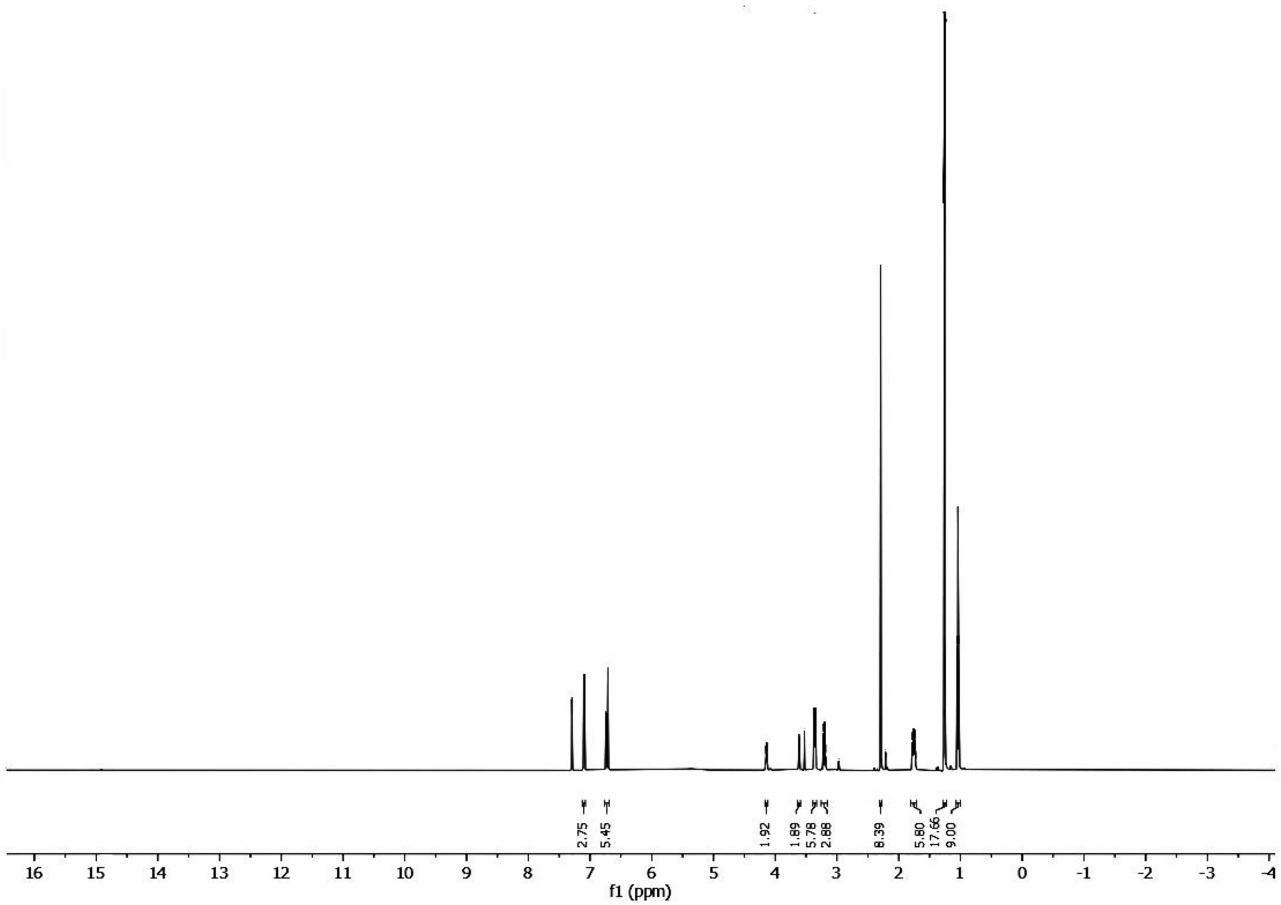

Figure S33: ^{1}H NMR of [N $_{3\,3\,3\,(2OH)}$$^{+}$][Br^{-}] : 2 Thymol HDES before extraction in KBr 30% aqueous solution

^{1}H NMR (400 MHz, $CDCl_3$) δ 7.06 (d, *J* = 7.7 Hz, 3H), 6.81 (d, *J* = 1.7 Hz, 6H), 4.15 – 4.03 (m, 2H), 3.60 – 3.50 (m, 2H), 3.38 – 3.26 (m, 6H), 3.20 (h, *J* = 6.9 Hz, 3H), 2.25 (s, 9H), 1.80 – 1.62 (m, 6H), 1.23 (d, *J* = 7.0 Hz, 18H), 0.98 (t, *J* = 7.2 Hz, 9H).

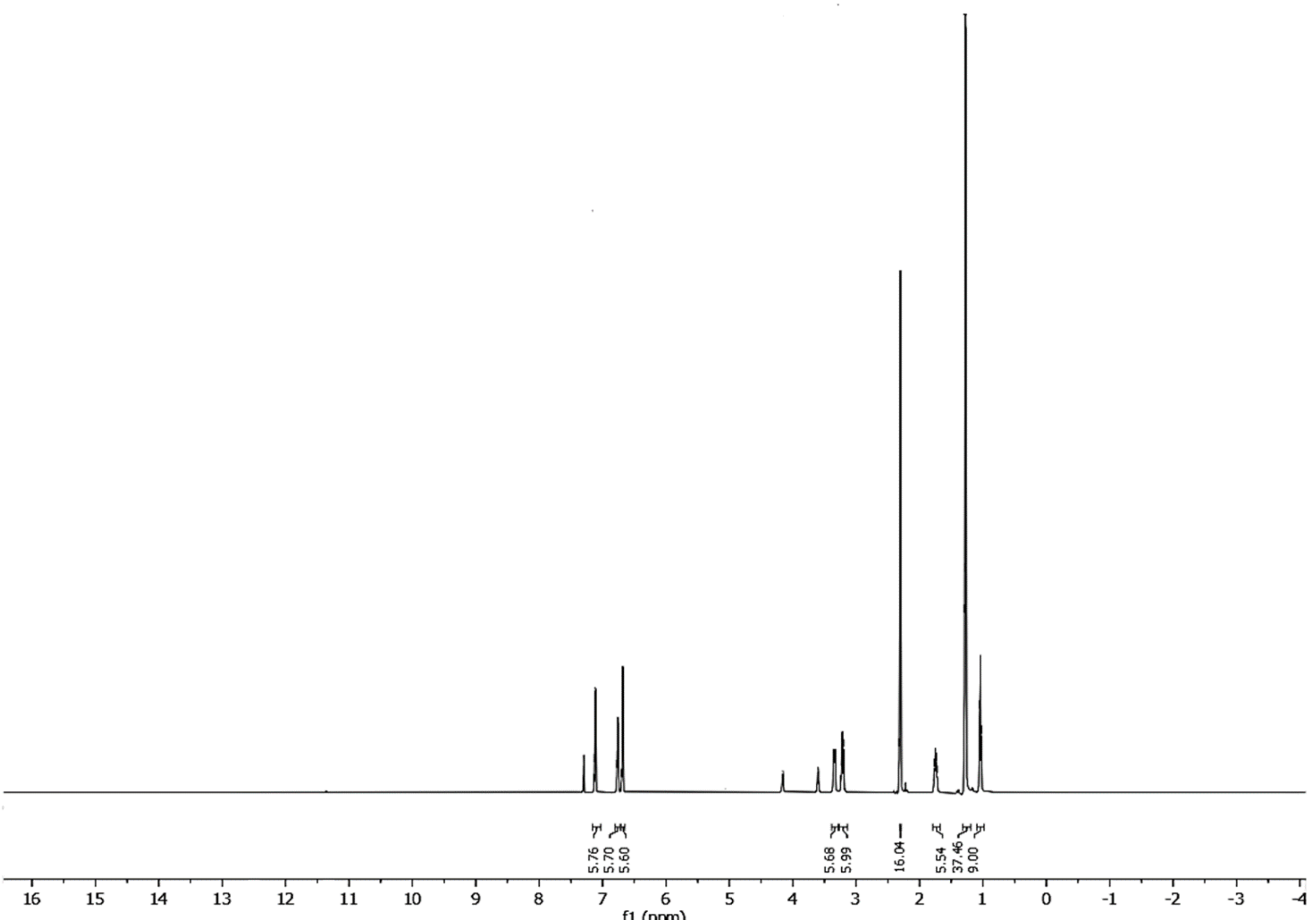

Figure S34: ^{1}H NMR of $[N_{3\,3\,3\,(2OH)}{}^{+}][Br^{-}]$: 2 Thymol HDES after extraction in KBr 30% aqueous solution

^{1}H NMR (400 MHz, $CDCl_3$) δ 7.10 (d, *J* = 7.7 Hz, 6H), 6.80 (d, *J* = 1.7 Hz, 6H), 6.67– 6.54 (m, 2H), 3.40 (m, 6H), 3.33 (h, *J* = 6.9 Hz, 6H), 2.30 (s, 16H), 1.79 – 1.65 (m, 6H), 1.34 (d, *J* = 7.0 Hz, 37H), 0.99 (t, *J* = 7.2 Hz, 9H).

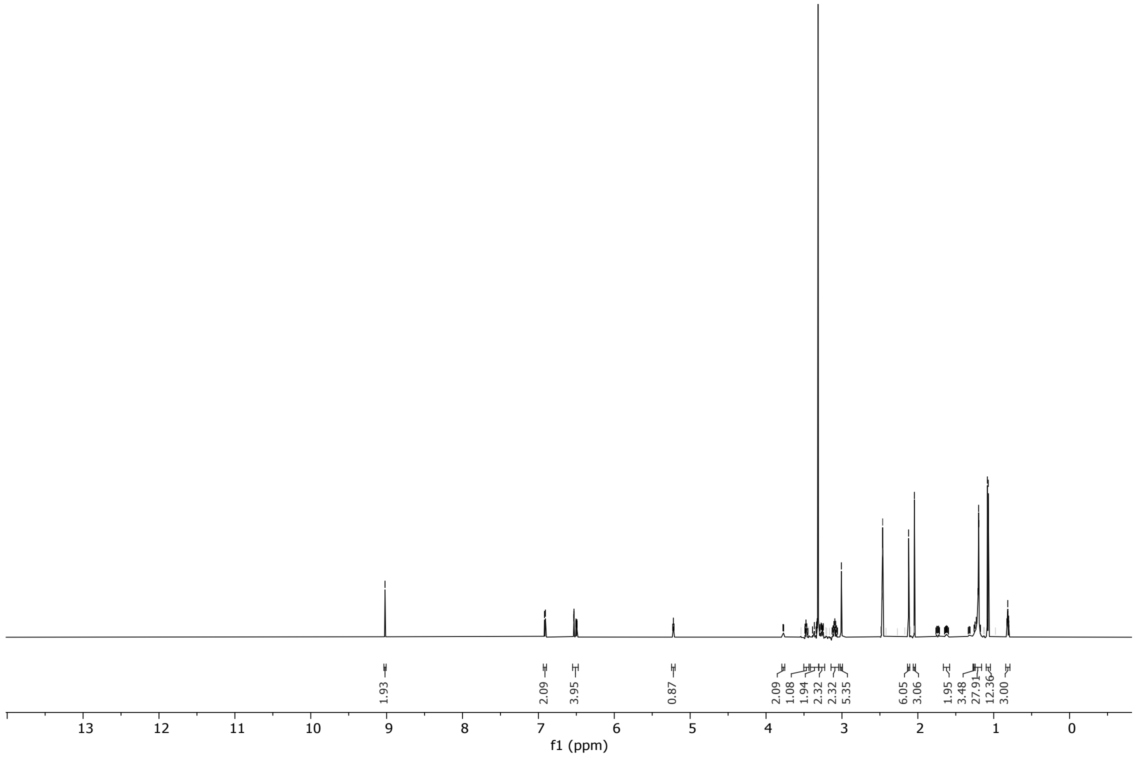

Figure S35: ^{1}H NMR of $[N_{1\,1\,16\,(2OH)}{}^{+}][Br^{-}]$: 2 Thymol HDES before extraction in KBr 30% aqueous solution

^{1}H NMR (600 MHz, DMSO- d_6) δ 9.02 (s, 2H), 6.91 (d, J = 7.7 Hz, 2H), 6.55 – 6.48 (m, 4H), 5.22 (t, J = 4.9 Hz, 1H), 3.77 (d, J = 5.1 Hz, 2H), 3.50 – 3.43 (m, 1H), 3.42 – 3.31 (m, 2H), 3.30 – 3.23 (m, 2H), 3.09 (p, J = 6.9 Hz, 2H), 3.01 (s, 5H), 2.12 (s, 6H), 2.05 (s, 3H), 1.62 (dq, J = 15.0, 7.7 Hz, 2H), 1.26 (s, 3H), 1.20 (t, J = 3.1 Hz, 28H), 1.08 (d, J = 6.9 Hz, 12H), 0.81 (td, J = 7.1, 0.9 Hz, 3H).

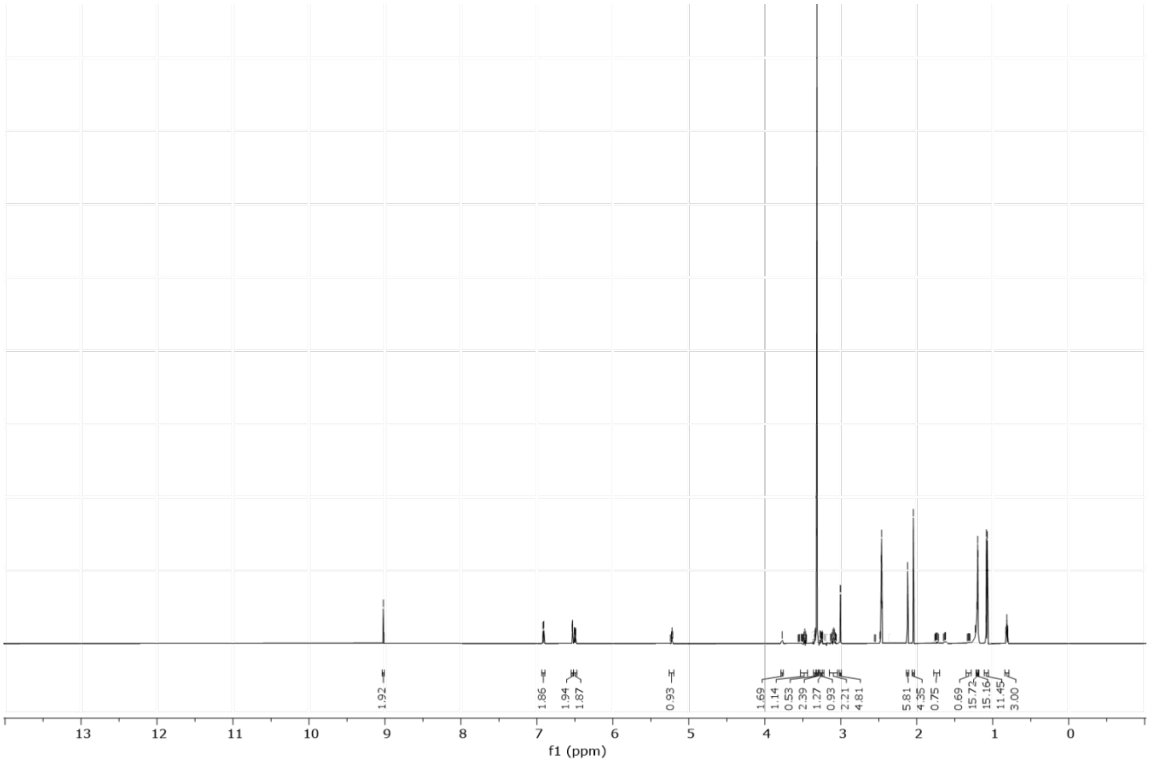

Figure S36: ^{1}H NMR of [N $_{1\,1\,16\,(2OH)}$$^+$][Br$^-$] : 2 Thymol HDES after extraction in KBr 30% aqueous solution

^{1}H NMR (600 MHz, DMSO- d_6) δ 9.02 (s, 2H), 6.91 (d, J = 7.7 Hz, 2H), 6.53 (dd, J = 1.7, 0.8 Hz, 2H), 6.52 – 6.48 (m, 2H), 5.22 (t, J = 5.0 Hz, 1H), 3.77 (s, 2H), 3.53 – 3.44 (m, 1H), 3.34 (d, J = 3.3 Hz, 1H), 3.32 (s, 2H), 3.26 (s, 1H), 3.25 (dd, J = 16.5, 4.0 Hz, 1H), 3.09 (dq, J = 14.0, 7.0 Hz, 2H), 3.01 (s, 5H), 2.12 (s, 6H), 2.05 (s, 4H), 1.74 (dt, J = 14.5, 6.8 Hz, 1H), 1.32 (q, J = 7.5 Hz, 1H), 1.21 (s, 16H), 1.20 (d, J = 1.3 Hz, 15H), 1.08 (d, J = 6.9 Hz, 11H), 0.84 – 0.79 (m, 3H).

References

(1) Dwamena, A. K.; Raynie, D. E. Solvatochromic Parameters of Deep Eutectic Solvents: Effect of Different Carboxylic Acids as Hydrogen Bond Donor. *J. Chem. Eng. Data* **2020**, *65* (2), 640–646. https://doi.org/10.1021/acs.jced.9b00872.

(2) Contreras, R.; Lodeiro, L.; Rozas-Castro, N.; Ormazábal-Toledo, R. On the Role of Water in the Hydrogen Bond Network in DESs: An Ab Initio Molecular Dynamics and Quantum Mechanical Study on the Urea–Betaine System. *Phys. Chem. Chem. Phys.* **2021**, *23* (3), 1994–2004. https://doi.org/10.1039/D0CP06078J.
